# Polymeric Systems as Hydrogels and Membranes Containing Silver Nanoparticles for Biomedical and Food Applications: Recent Approaches and Perspectives

**DOI:** 10.3390/gels11090699

**Published:** 2025-09-02

**Authors:** Alexandra Nicolae-Maranciuc, Dan Chicea

**Affiliations:** 1 Research Center for Complex Physical Systems, Faculty of Sciences, Lucian Blaga University of Sibiu, 550012 Sibiu, Romania; 2 Institute for Interdisciplinary Studies and Research (ISCI), Lucian Blaga University of Sibiu, 550024 Sibiu, Romania

**Keywords:** silver nanoparticles, natural polymers, synthetic polymers, hydrogels, membranes, antibacterial effect, tissue engineering, biomedical applications, food packaging

## Abstract

Silver nanoparticles (AgNPs) have garnered significant attention due to their potent antimicrobial properties and broad-spectrum efficacy against pathogens. Recent advances in polymer science have enabled the development of AgNPs-integrated hydrogels and membranes, offering multifunctional platforms for biomedical and food-related applications. This review provides a comprehensive overview of recent strategies for synthesizing and incorporating AgNPs into polymeric matrices, highlighting both natural and synthetic polymers as carriers. The structural and functional properties of these nanocomposite systems, such as biocompatibility, mechanical stability, controlled silver ion release, and antimicrobial activity, are critically examined. The focus is placed on their application in wound healing, drug delivery, food packaging, and preservation technologies. Challenges such as cytotoxicity, long-term stability, and regulatory concerns are discussed alongside emerging trends and safety paradigms. This work underscores the potential of AgNPs–polymer hybrids as next-generation materials and outlines future directions for their sustainable and targeted application in biomedical and food systems.

## 1. Introduction

In recent years, silver nanoparticles (AgNPs) have attracted growing attention due to their remarkable antimicrobial activity, which spans antibacterial, antiviral, and antifungal effects. These properties are largely attributed to mechanisms such as disruption of microbial membranes, generation of reactive oxygen species (ROS), and interference with essential cellular proteins [1,2]. Despite their effectiveness, the direct use of AgNPs faces limitations, including poor stability, potential toxicity to human cells, and the uncontrolled release of silver ions (Ag^+^) [3]. Incorporating AgNPs into polymer-based materials like hydrogels and membranes offers a promising solution to these issues, allowing for better control over silver release, enhanced dispersion, and improved compatibility with biological systems [4].

AgNPs can be incorporated into polymeric hydrogels. Hydrogels, three-dimensional, water-swollen polymer networks (both natural and synthetic), serve as excellent matrices for AgNPs due to their tunable porosity, mechanical properties, and biodegradability [5]. Recent studies have explored multiple polymer-hydrogel matrices, such as a kappa-carrageenan hydrogel incorporated with green-synthesized AgNPs, which demonstrated robust antibacterial efficacy and excellent cytocompatibility, tailored as a multilayer wound dressing [6]. Another approach uses carboxymethylcellulose hydrogels with in situ reduced AgNPs. The material revealed superabsorbent capacity (swelling ratio ≈ 1700%) and effective activity against *Escherichia coli* using silver nitrate (AgNO_3_) precursors [7]. Polymer–AgNPs nanocomposite hydrogels (e.g., carboxymethylcellulose–AgNPs systems) showcased promising anticancer and antimicrobial effects, highlighting their versatility in biomedical settings, as revealed by [8]. Such works highlight the dual functionality of hydrogels as both structural scaffolds and active antimicrobial platforms.

AgNPs can also be incorporated into polymeric membranes for food applications. In food science, the incorporation of AgNPs into polymeric membranes has gained attraction for active packaging, aimed at inhibiting spoilage and enhancing shelf life. For instance, biopolymer-based films, integrating AgNPs, significantly suppressed microbial growth in packaged fresh produce, dairy, and meat as reported by [9]. However, concerns arise from studies demonstrating AgNPs migration into foods, even with solid food items, raising safety and regulatory issues [10]. These findings illustrate the careful balance required between antimicrobial effectiveness and consumer safety. The selection of hydrogels, membranes, and scaffold-based polymeric systems as matrices for silver nanoparticles is driven by their complementary structural and functional properties. Each platform offers distinct advantages in tailoring the release kinetics, mechanical strength, and biological interaction of AgNPs-containing materials, particularly for biomedical and food-related applications [11]. Hydrogels are especially attractive due to their high-water content, soft tissue-like consistency, and ability to encapsulate both nanoparticles and bioactive agents within their crosslinked polymeric network. Their tunable swelling behavior and porosity enable responsive release mechanisms, often triggered by pH, temperature, or ionic strength, which are features valuable for wound healing, transdermal delivery, or localized antimicrobial action [5,6]. Membranes, by contrast, offer greater mechanical stability and barrier properties, making them ideal for food packaging, filtration, and implantable biomedical coatings. When loaded with AgNPs, polymer membranes not only prevent microbial contamination, but also serve as platforms for diffusion-controlled silver ion release. Biodegradable polymers such as poly(lactic acid) (PLA), chitosan, and cellulose derivatives are frequently employed to ensure environmental safety and biocompatibility [9].

In tissue engineering and regenerative medicine, scaffolds play a crucial role in mimicking extracellular matrices. The incorporation of AgNPs into these scaffolds adds an antimicrobial function, reducing infection risk during the critical early stages of cell proliferation and tissue regeneration [8]. These scaffolds can be molded into complex geometries and support cell adhesion, migration, and differentiation, especially when modified with biological cues or nanostructured features.

Several recent journal articles present original research into AgNPs-loaded polymeric systems, but our review focuses exclusively on the recent primary literature, aiming to clarify synthesis methods and applications of AgNPs–polymer composites, to highlight the structure–function relationships, especially in biomedical and food contexts, to examine performance metrics, toxicity assessments, and mechanistic insights from real-world applications. Moreover, this paper presents a discussion on the challenges and future perspectives, including regulatory landscapes and safety paradigms.

Previous reviews have primarily focused on broad overviews of AgNPs in nanomedicine, polymer nanocomposites in general [12], or food packaging applications [13]. However, just a few have systematically emphasized the role of polymeric systems specifically as functional carriers and stabilizers of AgNPs across biomedical and food-related contexts. This review distinguishes itself by critically discussing how polymer–AgNPs interactions govern cytotoxicity, release dynamics, and regulatory challenges, while also highlighting novel approaches such as 4D printing and sustainable recycling strategies. By integrating synthesis, performance, and application perspectives, this article aims to provide a multidisciplinary and application-oriented synthesis not fully addressed in earlier works.

## 2. Fundamentals of Silver Nanoparticles

The remarkable results in the field of nanotechnology led to an increased interest in researchers’ activity. Nanoparticles, a quite impressive domain of nanotechnology, possess important properties across various applications. The domain of nanoparticles is used for medical purposes, in biomedical engineering, food packaging, water treatment, environmental applications, and even electronics and automotive industries [14]. Nanoparticles exhibit various properties depending on their morphology and size; therefore, many synthesis approaches were proposed by researchers lately.

Silver nanoparticles are small particles with dimensions in a range of 1–100 nm with multiple properties. Their innovative properties have gained society’s attention; therefore, various studies and materials have been developed lately. AgNPs have superior chemical, physical, and biological properties, especially due to their structure and crystallinity in their nanometric form and size [15]. They are also known for their optical, electronic, and antifungal properties which make them great candidates in fields like optics, mechanics, pharmaceuticals, bioengineering, or environmental science [16,17,18]. In the last years, several studies have shown their benefits in biosensing applications. The AgNPs ability to improve electron-transfer activity and to amplify optical signals was the trigger for the development of various biosensors [19,20,21]. In these types of systems, AgNPs are usually combined with biological molecules or other metallic nanoparticles in order to improve their adhesion or their response. They are able to detect real-time activity and to monitor patients’ human changes in shorter periods compared to the classical investigation techniques. For instance, Geagea et al. [22] proposed a novel material based on AgNPs coated with zwitterionic polymers for an improvement of the colloidal stability of the nanoparticles in physiological medium, thus exposing the reaction conditions for AgNPs inside the human body [22]. Regarding the DNA modifications that appeared in time, AgNPs are an excellent tool for biosensors development. For instance, Huang et al. [23] proposed an electrochemical biosensor based on AgNPs-polydopamine graphene material for the precise detection of guanine and adenine. The results of tests performed show a very small detection limit for the material obtained in the laboratory compared to other biosensing tools [23].

In addition to biosensors, AgNPs are attached to various polymer matrices for a more controlled release or to improve their stability. Depending on the application, AgNPs can be found in soft materials, for example, hydrogels or scaffolds, but also in hard biomaterials, like those for bone regeneration or 4D printed materials. Despite their good optical and antibacterial properties, their stability in solution is an issue which is addressed lately by the scientific community. Their integration in hydrogels and membranes decreases the possibility of agglomeration, while their physical properties are maintained for longer periods of time. Many reviews and research articles state the impact of the size, shape, or morphology on AgNPs’ physical, optical, and biological properties [15]. The factors involved in the fabrication method together with the main components of the synthesis, reducing and stabilizing agents, should be chosen in relevance to the final application of the material.

In AgNPs fabrication, the reducing/stabilizing agents play the most important role since they have a strong impact on the final properties of the material. Therefore, in recent years, different synthesis techniques were proposed for AgNPs used as plasmonic particles or embedded in a polymeric matrix for further applications. The next subchapters will briefly discuss the main synthesis techniques for AgNPs and properties since the main focus of this work is to the AgNPs–polymer materials. Furthermore, we already published a review dedicated only to the AgNPs [24].

### 2.1. Synthesis Techniques for AgNPs

Currently, various techniques are applied to obtain AgNPs in the desired sizes and forms. The main three categories are physical, chemical, and biological methods. Physical and chemical syntheses are more classical, while biological methods, known also as green chemistry, are a more recent approach in which biological structures are used to obtain AgNPs.

**Figure 1:**
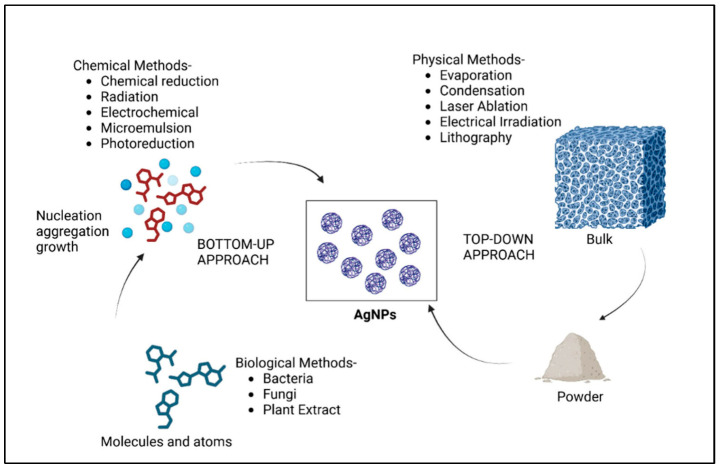
Main synthesis strategies for AgNPs: top-down (physical methods) and bottom-up (chemical and biological methods) approaches. Reprinted from an open-source article [14].

The formation of AgNPs can be obtained using top-down and bottom-up approaches [25], as described in Figure 1. The remarkable properties of AgNPs depend on their morphologies and configurations [26]; therefore, the chosen method of a proper synthesis is essential to obtain the perfect surface or structure. As the size and shape can determine the highest yield in a specific application, the synthesis method should be chosen in correlation to the research group resources and to the final destination of the material.

Top-down approach relies on the formation of AgNPs from a bulk material usually using physical methods and forces, such as crushing, grinding, or milling [26]. The physical techniques offer high purity and more uniformity to the nanoparticles; however, the necessity of complex equipment, high energy, and specialized research staff can be considered a problem. They do not have an environmental impact like chemical approaches since no chemicals are introduced in the reaction; however, the parameters are quite difficult to control and optimize [27].

The most applicable physical techniques are laser ablation [28], ball milling [29], and lithography [30], each with a specific application [4]. For instance, Mwenze et al. [31] synthesized AgNPs as a potential COVID treatment using laser ablation in liquid. The physical–chemical characterization showed specific UV-VIS peaks for AgNPs, while SEM confirmed the 16–30 nm sizes suitable to be used as drug carriers for hydroxychloroquine in COVID-19 treatment [31]. Abdulraheem et al. [32] confirmed the ability to obtain nanometric sizes for the AgNPs using the same technique. The research group proposes the fabrication of 12–20 nm AgNPs using a pulsed laser ablation in liquid with high precision in distilled water. The spectroscopy analyses confirmed the specific peaks for AgNPs, while TEM indicated a spherical form for AgNPs, the most suitable for biomedical applications [32]. Kováčová et al. [33] revealed an interesting work in which ball milling was mixed with a green synthesis approach to obtain AgNPs. The study used different AgNO_3_:plant mass ratios for the mechanical approach and *Thymus vulgaris* L., *Sambucus nigra* L., *Thymus serpyllum* L. plants as reducing agents. The synthesis was performed based on a simple one-step solid-state approach, obtaining nanosized silver nanoparticles with superior antibacterial properties for all plants tested [33].

Bottom-up approaches involve a different technique for AgNPs fabrication. Based on chemical and biological syntheses, bottom-up methods rely on molecules and atoms which, through the nucleation and aggregation process, achieve the nanometric size of the particle. This approach forms complex aggregates starting from metallic salt precursor with the help of reducing agents and stabilizing reagents. The reduction agents can be a chemical reagent or an active compound from a biological source. Its role is to reduce the metallic precursor into final nanoparticles stabilized through the stabilizing agent which will ensure a stable surface of the final particles. This stability will avoid aggregation and will maintain their efficiency for further applications. Chemical and biological syntheses offer high yield of reaction, they are cost-effective, they do not need complex equipment, and they can be obtained with limited resources, especially for the biological methods where the sources are natural and can be easily procured. As is illustrated in the figure above, the common chemical routes are chemical reduction [34], microemulsion [35], electrochemical [36], or photoreduction [37]. Various studies were performed to obtain AgNPs using a chemical reduction since it is the most accessible alternative route. Quintero-Quiroz et al. [38] revealed the importance of the process optimization in AgNPs synthesis varying many parameters: concentration of precursors/agents and the pH of all reactions. The study proved the sensibility of AgNPs to all these parameters, leading to the conclusion that average size, polydispersity, and yield reaction can be easily controlled. The work also proved differences in antibacterial effectiveness depending on parameter reaction and bacterial strains [38]. Haider et al. [39] exposed the influence of stabilizing agents in the antibacterial activity of AgNPs. The study revealed a higher inhibition zone in the disk diffusion antibacterial test for AgNPs coated with trisodium citrate for *Staphylococcus aureus* strain [39]. The importance of chemical reduction optimization was also reported by Horne et al. in a study aimed to enhance the nanoparticles’ surface-enhanced Raman scattering (SERS) sensitivity [40].

**Figure 2:**
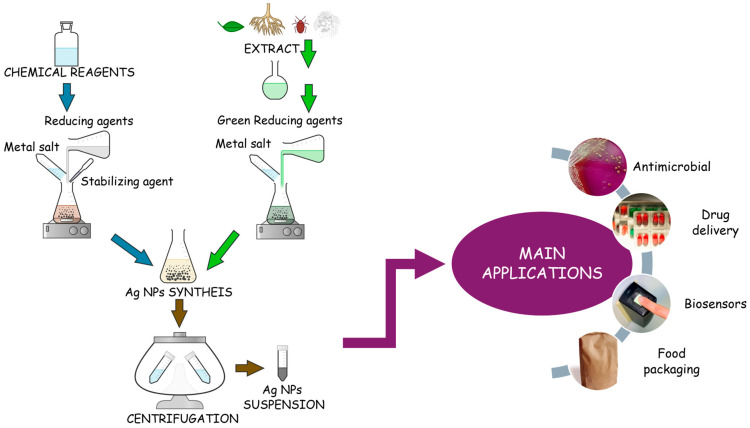
Schematic diagram illustrating the comparison between chemical reduction (starting from chemical reagents to synthesize AgNPs) and green synthesis (based on plants, micro-organisms, and bacteria as reducing agents for AgNPs synthesis) together with the major applications of AgNPs.

Green synthesis, the innovation of classical chemical synthesis, is an eco-friendly approach in which biological extracts or micro-organisms are used as bioreducing agents in AgNPs formation. Figure 2 illustrates the main differences between chemical routes and green synthesis, with the accent also on the major application of AgNPs fabricated. In both techniques, the metal source is similar, usually a silver salt like AgNO_3_; however, the reducing agent is different. Both reactions involve the reduction in ions to atoms which are further nucleated into particles [26]. The salt and the reducing agent concentrations govern the size and shape of the final AgNPs in both types of reductions. Some chemical reagent examples include sodium borohydride/citrate [41], while green agents can be considered any plant extract, bacteria, or fungi [42]. Lately, a large quantity of studies have been performed in the direction of green synthesis since it is cost-effective, eco-friendly, and a technique with unlimited resources. For instance, Zahran et al. [43] proposed the biosynthesis of AgNPs using *Ficus sycomorus* leaves to obtain an electrochemical sensor for detection of bromocresol green in river water. Synthesis showed good chemical stability leading to AgNPs with spherical form and an average size of 20 nm [43]. Khan et al. [44] reports the fabrication of antibacterial AgNPs using *Salvia sclarea* leaf extract in the green synthesis. The results proved a direct dependency between Ag concentration and the level of antibacterial effectiveness [44].

### 2.2. Physicochemical Properties of AgNPs

The versatility of AgNPs is sustained by their incredible optical, electronic, and biological properties. These nanoparticles exhibit surface plasmons resonance (SPR) in which electrons are conducted at specific wavelengths depending on the size and shape of the particles. This important feature is the basis in biosensors development and imaging techniques; therefore, the addition of AgNPs in such smart devices has increased a lot lately [45]. According to the literature, spherical AgNPs demonstrate one surface SPR, while triangular AgNPs can exhibit three SPR bands due to the dipole and quadrupole plasmon resonance [45]. In addition to the shape, the size can also influence these SPR bands. Typically, for a colloidal solution, with AgNPs under 100 nm, the localized-SPR (LSPR) peaks are in a range of 380–460 nm, showing the dispersity behavior of the solution [46].

For biological activity, the focus of this review, the size and the shape are also the main properties to be considered in the synthesis process. AgNPs can be found in various shapes like spherical, triangular, cubic, and hexagonal [4]. A study performed by El-Zahry et al. [47] revealed a comparison between three different AgNPs, spherical, triangular and hexagonal, with an average size of 40 nm. The results showed that the highest antimicrobial effect for *Escherichia coli* was established for hexagonal AgNPs [47]. According to the literature, the antibacterial effect of AgNPs is also strongly influenced by their size. The size-dependent efficacy of AgNPs is sustained by many reports which suggest a stronger antibacterial effect with a lower minimum inhibition concentration (MIC) [48,49]. Secario et al. [50] proved the efficiency of green-synthesized AgNPs against *Staphylococcus aureus* strain. The study involved two different green compounds, green tea leaf and cassia seed, as reducing agents. The results showed smaller nanoparticles for cassia seed, 12 nm compared to 25 nm, and lower minimum inhibitory concentration for these smaller nanoparticles [50]. Another study which sustains this affirmation was reported by Ji et al. [51]. They managed to synthesize AgNPs using a thermos-sensitive copolymer as a stabilizing agent. The chemical reduction proposed led to an innovative strategy for AgNPs with high antibacterial effect. The biological tests performed in this study, antibacterial effect, intracellular ROS test, and detection of bacterial membrane integrity showed the better antimicrobial activity for the smallest AgNPs of just 1.59 nm compared to 4 nm. Also, for this ultrasmall nanoparticle, the MIC was the lowest, with a value of 4.05 μg/mL [51].

Table 1 illustrates some examples for AgNPs sizes depending on the synthesis routes. Also, there are mentioned some key aspects related to the material and the final results regarding the antibacterial effect.

### 2.3. Antimicrobial and Biological Activity

For polymeric systems, the biological mechanism of AgNPs is based on the release of the particles and ions from the matrix based on their diffusion rate. The incorporation of AgNPs in polymer matrices such as hydrogels, scaffolds, membranes, or films in a promising strategy to improve their stability and to create support for their better delivery. The antimicrobial and biological activity of AgNPs described further is similar once they are in contact with tissues, cultures, or biological structures regardless of their encapsulation method (in suspensions or incorporated into polymers). Many studies in the literature claim an improvement in the stability and release of AgNPs and Ag^+^ once AgNPs are embedded in a polymer matrix [58]. The polymers offer support for regeneration and reconstruction, while particles and Ag^+^ are dissolved to the wound level at the same time. Hu et al. [59] proved in a recent study the successful synthesis of a hybrid hydrogel material based on triple-helix β-glucan and AgNPs for wound healing. The results showed a broad antimicrobial activity against four bacteria strains, indicating significant bacteria inhibition for both Gram-positive and Gram-negative strains. The study also sustains the idea that Ag^+^ are released in addition to the AgNPs incorporated in the hydrogel, leading to a better understanding of this mechanism. To analyze the release of Ag^+^ from the hydrogel, they studied the hydrogel swelling properties and concluded an increase in Ag^+^ release with the increasing immersion time in PBS. Most of the ions were released in three days, a perfect timeline to stop the bacteria development in the injured areas [59]. Another study which sustained the involvement of both AgNPs and Ag^+^ was conducted by Aldakheel et al. [60]. They fabricated a hydrogel based on chitosan and PVA for wound healing applications. The results showed various release rates for Ag^+^ depending on the timeline; however, they concluded that by increasing AgNPs concentration and increasing the release time, the amount of Ag^+^ can increase from 5.9 μg/mL up to 19 μg/mL in two days. The antibacterial effect was also confirmed by the research group [60].

**Figure 3:**
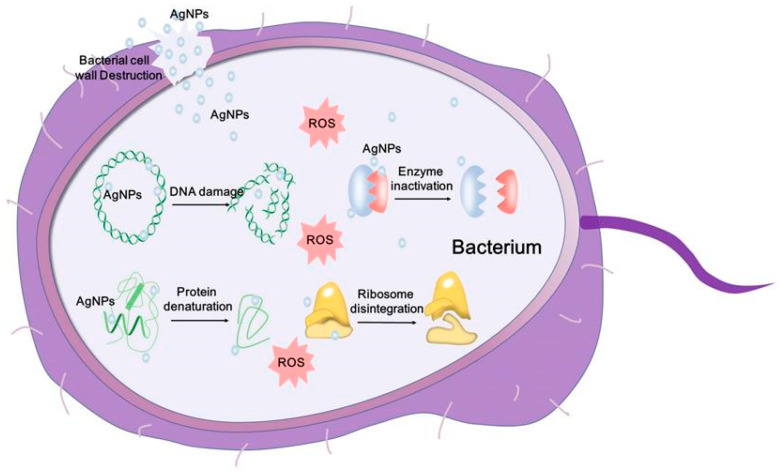
Schematic illustration for AgNPs mechanism against bacteria: membrane penetration, AgNPs release, and ROS production together with biochemical modifications. Reprinted from an open-article source [61].

The mechanism of AgNPs has been studied for years; however, the complexity of their cytotoxic effect is still unclear. Lately, it has been observed that AgNPs penetrate the bacterial membrane first, as can be observed in Figure 3 which shows the modifications that appeared inside the bacteria.

Once the membrane is disrupted, the cell begins to function unproperly, and the ROS appears to lead to enzyme inhibition and DNA damage. More specifically, upon endocytosis or direct penetration of the membrane, AgNPs will undergo oxidation by O_2_ or other biochemical molecules releasing Ag^+^. This Ag^+^ form will create bonds with S and N, leading to free radicals and complexes with sulfur-proteins and peptides [62]. These resistant complexes induce modifications in cell metabolism, especially leading to a decrease in the yields of biochemical reactions. The higher the amount of Ag^+^ in the cell, the more cellular dysfunction increases. The ROS production is stimulated by this oxidation process, increasing the oxidative stress of the cell [63]. Together with ROS, several signaling pathways such as MAPK pathway or mTor pathway are also triggered [63,64]. Being part of an essential cellular mechanism such as inflammation, proliferation, and cell death [63], the activation of such pathways by AgNPs, or directly by ROS, will increase the chances of side effects. In addition, in the presence of AgNPs, the endoplasmic reticulum stress, another cell mechanism defense, is activated while protein folding is reduced [65]. The incorrect protein folding and high oxidative stress that appeared inside the cell led in most cases to apoptosis or necrosis. Lately, the scientific community has also proved the implication of mitochondria in this toxic effect of AgNPs. According to many reports, including [66,67], mitochondria plays an important role in cellular metabolism modifications and even in cell death since AgNPs see them as a sensitive target upon membrane penetration. Li et al. [68] studied the uptake and cytotoxicity of silver nanowires in keratinocytes and macrophage cells evaluating the ROS production and the mitochondrial activity. Based on toxicological assays and fluorescent microscopy, the results proved a higher impact of silver nanowires in mitochondrial membrane disruption in case of keratinocytes compared to macrophages [68]. Therefore, all these mechanisms proved to be cell-dependent, showing that the cell type is a crucial factor in assessing the nanomaterials’ toxicity.

In case of bacteria, Ag^+^ from AgNPs can play the role of cofactors for bacterial enzymes involved in ROS production [1]. Through the appearance of ROS, the oxidative stress increases, and the DNA suffers modifications while the replication is inhibited, leading to the apoptosis of the bacterial cell [61,69]. These mechanisms and the antimicrobial effect of AgNPs are the basis of antibacterial materials research. Salayová et al. [70] synthesized AgNPs using five different plants and green chemistry. The synthesis process was rigorously monitored, with observable color changes and favorable outcomes confirmed by UV-VIS spectroscopy. Nanoparticle characterization was performed using a range of analytical techniques. XRD confirmed the successful reduction, while FT-IR spectroscopy indicated interactions between plant-derived bioactive compounds and the nanoparticles. The antimicrobial potential of the AgNPs was assessed via the agar diffusion method, demonstrating significant antibacterial activity against five pathogenic bacterial strains: *Listeria monocytogenes*, *Staphylococcus aureus*, *Pseudomonas aeruginosa*, *Escherichia coli*, and *Salmonella enterica*. Notably, the strongest antibacterial effects were observed in AgNPs synthesized using *Brassica nigra* and *Lavandula angustifolia* extracts [70]. The study conducted by Shanmugam et al. [71] highlights the versatility of the green synthesis approach for producing nanoparticles with controlled size and morphology, suitable for medical, antioxidant, and antimicrobial applications. In their work, AgNPs were synthesized using a natural extract from *Allium cepa* var. *aggregatum*. Antibacterial activity, evaluated via the agar diffusion method, revealed efficacy against both *Staphylococcus aureus* and *Escherichia coli* bacteria [71].

## 3. Polymeric Matrices for AgNPs Incorporation

Polymeric matrices are classified as natural or synthetic depending on the polymer type chosen for the final application. Natural polymers are known for their abundance in nature, their excellent biocompatibility and biodegradability, and their ability to stimulate tissue regeneration in the implantation area. Nevertheless, they are easy to work with and, in most cases, they can be combined with multiple other materials to improve the final properties. On the other hand, synthetic materials are mainly fabricated in the laboratory. Their main role is to offer better stability and mechanical properties for the final material. In biomedical and food applications, they are combined with natural polymers to assure both high biocompatibility as well as good mechanical and thermal stability in time.

### 3.1. Natural Polymers

Natural polymers are used in biomedical applications due to their natural state. They are components of biological systems with the purpose of restoring the essential functions of injured areas. In addition to the well-known biomedical applications, natural polymers found activity also in the food industry, especially in food packaging. Natural polymers possess many advantages; however, their major benefit remains the high biocompatibility. They have minimal side effects, increase the regeneration in tissues and organs, and improve the material tolerance of the human body. Even though they offer such excellent biological properties, natural polymers have poor stability and mechanical properties. They are stable in specific media for short periods of time, and they degrade very fast. They are able to offer support for minimal injuries; however, for deeper wounds or more complex situations, the addition of a synthetic polymer is necessary.

**Figure 4:**
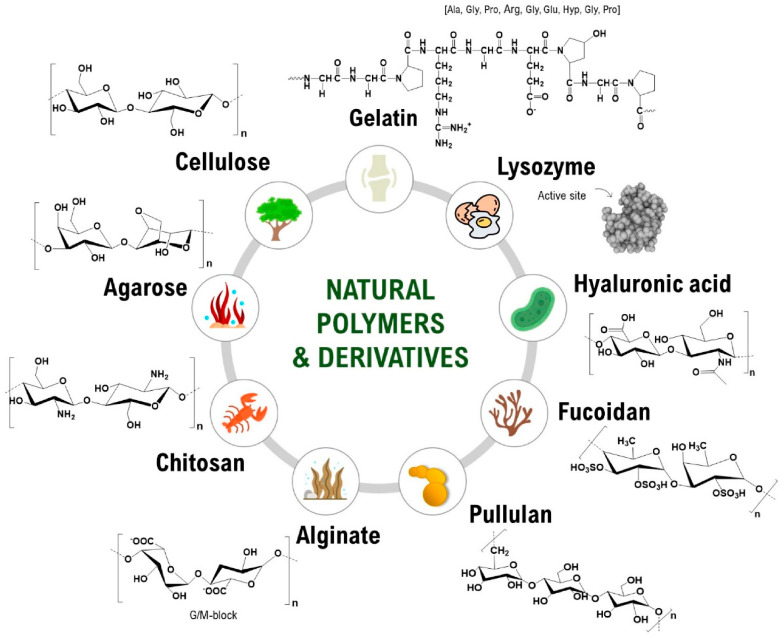
Various examples of natural polymers for biomedical and food applications together with their chemical structure. Reprinted from an open-source article [74].

Natural polymers are extracted from organic sources, usually from micro-organisms, plants, animals, or algae [72]. The unlimited resource, their diversity, and the similarity with the extracellular matrix are also advantages of this class of materials since the immunological response and toxicity are usually absent once they are in contact with the body’s areas. They possess high degradability, depending on the extraction process and the media in which they are maintained; therefore, they can participate in the regeneration process, offering comfort to the patient. Proteins or polysaccharides, found in various living things or natural resources, are considered natural polymers, being part of this class [73]. Some examples of natural polymers are detailed in Figure 4, together with their chemical structure [74].

Cellulose, chitosan, collagen, gelatin, and alginate are the most studied polymers for biomedical applications. Their very high similarity with natural tissues and their ability to form hydrogels, dressings, or scaffolds led to the fabrication of many composites with AgNPs. In most cases, all these polymers offer support for easier administration of AgNPs. Cellulose, a linear chain of 2 d-glucose units linked by β-1-4 glycosidic bonds [75], is known as the most abundant natural polymer from the planet, being naturally secreted by plants and bacteria in a pure form [74]. In recent decades, various forms of cellulose have been presented, including nanocellulose implemented usually in nanocomposites [76,77]. Its impressive mechanical and thermal stability is used to reinforce a material, being usually found together with another natural or synthetic polymer. Chitosan, a linear polysaccharide composed of β-(1–4)-linked D-glucosamine and N-acetyl-D-glucosamine units randomly mixed, belongs to the polysaccharides class. The main chitosan sources are marine organisms or micro-organisms; therefore, it is an important abundant polymer found in nature [78]. It has excellent biocompatibility and biodegradation for tissue engineering applications. Its association with AgNPs allows the formation of an antibacterial hydrogel or dressing for wound infections. Collagen, the most abundant protein from our body, is another natural polymer considered an excellent candidate for tissue regeneration [79]. Biomaterials based on collagen show high biocompatibility and an impressive regenerative effect for the cells. They are doped with AgNPs for antibacterial applications since they inhibit bacteria proliferation, while offering a faster healing compared to other natural polymers. Stable collagen fibers used in nanocomposites can be obtained through a chemical crosslinking step in which the final AgNPs-based material is reinforced [80].

Many reports state the fabrication of hydrogels and membranes with natural polymers and AgNPs. For instance, Lei et al. [81] fabricated a wound-dressing hydrogel based on chitosan which improved the hemostasis and the antibacterial effect once the AgNPs were embedded. The biological test showed a fast and improved healing of skin wounds in rats infected with *Staphylococcus aureus* [81]. Diniz et al. [82] proposed a composite hydrogel based on alginate and gelatine doped with AgNPs as biocompatible device for easy administration and low ecological impact. The cytotoxic effect was proved to be absent for fibroblasts while the antibacterial activity was confirmed for *Staphylococcus aureus* and *Pseudomonas aeruginosa* strains [82]. Li et al. [83] also revealed the optimization of a natural hydrogel film based on chitosan and sodium alginate with tannins and AgNPs for antibacterial applications. The study revealed good flexibility and good swelling properties of the hydrogel film. Furthermore, the hemolysis analysis performed on rabbit blood showed excellent effect, while the antibacterial effect revealed a total inhibition of bacteria at pH 8 and 10 [83].

### 3.2. Synthetic Polymers

Synthetic polymers are defined as structures obtained in the laboratory with the purpose of improving the final properties of the material in which they are introduced [84]. Synthetic polymers are classified in thermoplastic and thermoset polymers [72]. They are found in biomedical devices and applications due to their good mechanical properties. They are stable, controllable, and, usually, they accelerate the reactions in which they are involved. Synthetic polymers are also found in textile and food packaging industries, in medicine, or in automotive industries since they are also flexible and resistant to higher temperatures [85]. In biomedical applications, they are involved in tissue engineering, drug delivery, anticancer applications, and even in radiology. Good compatibility, tunable properties, superior mechanical properties, surface modification, and easy process in the laboratory are also features for which synthetic polymers have been studied for years [86]. Indeed, compared to the natural polymers, their integration in the body is achieved with less tolerance; however, synthetic polymers are developed as biocompatible, and sometimes also biodegradable, polymers. Some examples are poly(vinyl alcohol) (PVA), polyethylene (PE), poly(ethylene glycol) (PEG), poly(lactic-co-glycolic acid) (PLGA), PLA, polytetrafluoroethylene (PTFE), polyamides (PA), poly(vinyl chloride) (PVC), and many others [72].

Mačák et al. [87] fabricated a nanocomposite based on AgNPs and PVA as an eco-friendly material with antimicrobial activity [87]. Teper et al. [88] revealed the fabrication of a promising hybrid material by synthesizing AgNPs directly on poly(N,N′-dimethylaminoethyl methacrylate) and hydroxyl-bearing poly [oligo (ethylene glycol) methacrylate layers. The final antibacterial effect of the nanolayers created proved a strong antibacterial effect on both Gram-positive and Gram-negative bacteria strains, leading to promising antibacterial coatings for biomedical applications.

To summarize this chapter, we can conclude that natural polymers excel in biocompatibility and inherent bio-functionality but are less stable, while synthetic polymers offer superior mechanical strength, scalability, and release control. Blended systems often achieve the best compromise.

## 4. Hybrid AgNPs–Polymer Systems

**Figure 5:**
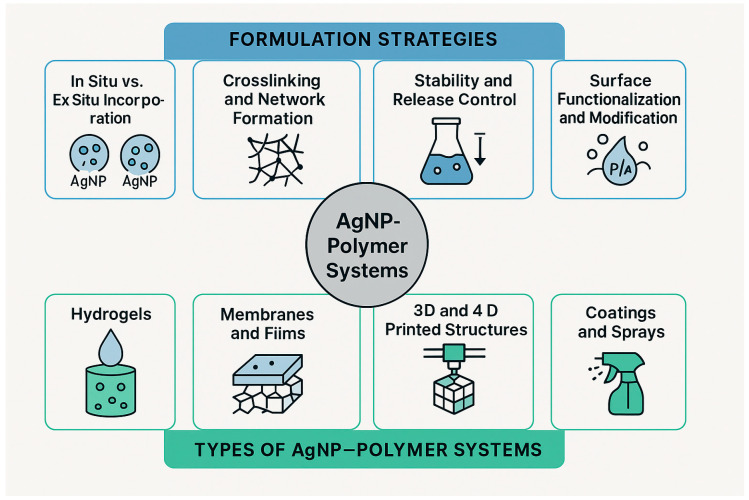
The formulation strategies and types of AgNPs–polymer systems taking into consideration the nanoparticle incorporation method, the crosslinking strategy, and the stability and the surface functionalization of AgNPs in hydrogels, membranes, 3D/4D printed structures, and coatings/sprays.

In the following sections, we examine each category of AgNPs–polymer systems, namely hydrogels, membranes, scaffolds, 3D/4D printed structures, and coatings or sprays by discussing their formulation and functional characteristics. For each system, we focus on key aspects, including the mode of nanoparticle incorporation (in situ versus ex situ), strategies for crosslinking and network formation, mechanisms governing stability and controlled silver release, and surface functionalization approaches, as it is described in Figure 5.

### 4.1. Hydrogels

Many studies favor in situ AgNPs synthesis, where silver ions are reduced directly within the hydrogel matrix during gelation, resulting in uniform dispersion and stable integration. For instance, the work presented in [89] reports on developing an injectable hydroxypropyl methylcellulose/hydroxyapatite hydrogel in which in situ formation of approximately 3–17 nm AgNPs occurred without added reducing agents. This platform exhibited excellent antimicrobial activity and achieved approximately 94% wound closure in a murine burn model. In contrast, ex situ methods involve synthesizing AgNPs separately and then incorporating them into the hydrogel. Although this allows tighter control over particle size and shape, it risks aggregation and inconsistencies in distribution unless properly stabilized, as seen in citrate- or PEG-coated AgNPs-hydrogel systems [90].

Hydrogel architecture is heavily influenced by crosslinking chemistry, which determines porosity, mechanical strength, and swelling, which are all key to drug and silver release profiles. For example, the hydrogel proposed in the study of Qiu et al. [89] relied on hydrogen-bonding networks for moderate stiffness and porous structure [89]. In another approach, chitosan–PVA hydrogels underwent microwave-assisted in situ reduction, which concurrently crosslinked the network and synthesized approximately 22 nm AgNPs, ensuring sustained silver release [90]. Mechanical stability and controlled Ag^+^ release are essential to balance antimicrobial action against potential cytotoxicity. Hydrogels responsive to pH, ionic strength, or temperature allow finely tuned, on-demand release. For example, carrageenan-based systems exploit ionic changes to modulate Ag^+^ diffusion [91].

To enhance biological interactions and nanoparticle stability, hydrogels are often chemically modified. PEGylation provides anti-fouling properties, while peptide conjugation encourages cell adhesion. Green hydrogels, like those proposed by Aldakheel et al. [90], use plant-based reducing agents (e.g., garlic extract) both to synthesize AgNPs and impart biocompatibility, promoting healing in diabetic wound models [90].

### 4.2. Membranes and Films

Membranes and films in AgNPs–polymer systems are widely used in antimicrobial coatings, filtration, wound care, and packaging.

To achieve in situ incorporation, silver ions are loaded into the polymer precursor and reduced chemically/thermally, generating AgNPs within the matrix. These yield well-dispersed, narrowly sized nanoparticles owing to templating effects and stabilized growth [92]. Opposed to these types of procedures, for ex situ alternatives, the AgNPs are synthesized separately and then blended into the polymer solution or deposited on the surface, thus allowing a tight control of NPs size and shape, but risks aggregation and weaker integration [93,94]. Table 2 illustrates a comparation of the in situ with ex situ incorporation methods for AgNPs.

The structural design of nanoparticle-loaded polymer membranes is critically influenced by the crosslinking strategy employed. Physical crosslinking techniques, such as freeze–thaw cycling or ionic gelation, are particularly effective for hydrophilic, water-soluble matrices like PVA or alginate. These methods avoid the use of chemical agents and are frequently utilized in systems where biocompatibility and minimal processing are desired, as demonstrated in alginate–AgNPs composite films [95]. In contrast, chemical crosslinking using agents such as glutaraldehyde or genipin offers superior control over network rigidity and durability. These agents enhance the mechanical integrity of the membrane and contribute significantly to the long-term retention of AgNPs, ensuring membrane robustness under operational conditions [96].

A more versatile approach involves the development of interpenetrating polymer networks, where two or more polymer networks coexist in a physically entangled but chemically independent structure. This configuration enables fine-tuning of nanoparticle immobilization and membrane permeability, providing a customizable platform for controlled AgNPs distribution. An example includes PVA/polyacrylic acid/graphene oxide–Ag composite membranes, which exploit the synergistic properties of the constituent polymers to improve both mechanical strength and nanoparticle stability [97].

The overall crosslink density and network architecture are critical design parameters, directly impacting membrane porosity, mechanical behavior, and the release kinetics of embedded nanoparticles.

Ensuring the colloidal stability of AgNPs within a membrane matrix is essential for sustained antimicrobial performance. This is often achieved through in situ reduction techniques, where silver ions are reduced directly within the polymer structure in the presence of stabilizing agents. Coatings with materials such as PEG or polydopamine are commonly applied to further enhance dispersion stability and prevent nanoparticle aggregation. Additionally, control over pore size and membrane architecture plays a central role in regulating Ag^+^ release. Membranes engineered with narrow or selectively distributed pores, or those incorporating multilayered structures and diffusion barriers, have demonstrated sustained release profiles [98]. These features are particularly advantageous in applications requiring long-term antimicrobial action.

In more advanced systems, stimuli-responsive release mechanisms have been implemented. Here, polymers sensitive to environmental triggers, such as pH or temperature, modulate the release of Ag^+^ in response to changes in the surrounding medium. For instance, wound environments characterized by pH fluctuations can be used to initiate silver ion release, thus enhancing therapeutic precision. The oxidative dissolution of AgNPs remains the primary mechanism for silver ion generation, enabling continuous antimicrobial activity through controlled ion flux [98].

The effective immobilization of AgNPs on membrane surfaces is often facilitated by chemical surface modification. The introduction of functional groups such as hydroxyl (–OH), carboxyl (–COOH), and amine (–NH_2_) onto the membrane surface, either inherently present in materials like graphene oxide or introduced through chemical treatment, enhances the binding affinity for AgNPs, leading to improved distribution and stability [99]. Techniques such as plasma surface activation and polydopamine coating further increase surface reactivity, offering additional sites for nanoparticle attachment and improving adhesion under operational conditions [100].

### 4.3. Scaffolds

Three-dimensional scaffolds are formed from natural or synthetic polymers and offer structural support, enhance cell migration, and serve as reservoirs for bioactive agents. Incorporation of AgNPs into these platforms promotes antimicrobial activity but demands precise control over synthesis, stability, and release dynamics.

In situ incorporation involves generating AgNPs within the scaffold during fabrication, ensuring uniform dispersion and minimizing nanoparticle aggregation. The study presented in [101] reports on UV-reduced AgNPs in 3D printed collagen scaffolds. The authors mention that AgNPs (~10–28 nm) were formed directly within collagen matrices and exhibited enhanced thermal stability and swelling, along with strong antibacterial activity against *Escherichia coli* and *Staphylococcus aureus* [101]. The in situ addition of AgNPs is strengthening the final scaffold properties. Another example consists of polydopamine-mediated AgNPs in electrospun PLGA/polycaprolactone (PCL) scaffolds. Polydopamine coating enables in situ reduction in AgNPs while it is coupled with collagen immobilization. The result is a scaffold with osteogenic signaling and antimicrobial efficacy against *Staphylococcus aureus* and *Streptococcus mutans* both in vitro and in vivo [102]. Another study performed by Radhakrishnan et al. [103] revealed the fabrication of a scaffold using chemical-reduction on plasma-activated PCL nanofibers. Argon plasma treatment introduces hydrophilic groups, enabling NaBH_4_-mediated AgNPs synthesis at around 92 nm [103]. These scaffolds display a burst release (12 h) followed by sustained release over approximately two weeks, with effective antibacterial activity [103].

Another route for incorporating AgNPs is ex situ incorporation. This procedure entails embedding pre-synthesized and stabilized AgNPs into pre-formed scaffolds, offering enhanced control over nanoparticle characteristics. Citrate-capped AgNPs on collagen-coated PCL nanofibers is an example of such scaffolds. Collagen-coated PCL mats were functionalized with pre-synthesized silver nanoparticles (~20 nm), resulting in a composite that exhibits broad-spectrum antimicrobial activity against *Staphylococcus aureus*, *Pseudomonas aeruginosa*, and drug-resistant *Enterococcus* species, while preserving biocompatibility, as reported by [104].

The physical properties of AgNPs-loaded scaffolds, particularly their mechanical strength, porosity, and nanoparticle retention efficiency, are highly dependent on the crosslinking strategies employed during fabrication. One effective approach involves polydopamine-induced crosslinking, which has shown notable results in scaffolds composed of PLGA/PCL. In these systems, polydopamine serves a dual function: it facilitates the attachment of AgNPs to the polymer surface while simultaneously promoting crosslinking within collagen networks from the organ. This dual action leads to enhanced mechanical integrity and improved cellular compatibility, making the scaffolds more suitable for tissue engineering applications [102].

A second method combines photopolymerization with in situ nanoparticle synthesis, where UV exposure not only initiates the formation of AgNPs within the collagen matrix but also increases the crosslink density of the scaffold. This simultaneous process reinforces the structural stability of the material and slows its degradation rate, contributing to prolonged functionality in biological environments [101].

Additionally, scaffolds based on polymer–mineral composites present a promising avenue for achieving multifunctional properties. For example, integrating PCL with bioactive calcium phosphate matrices results in a mechanically robust scaffold with osteoconductive characteristics. The inclusion of AgNPs, whether incorporated during synthesis or added post-fabrication, further enhances the system by introducing antimicrobial functionality alongside bone-regenerative capacity, demonstrating the potential for synergistic therapeutic performance [105].

The controlled release of silver ions is a critical design parameter in AgNPs-based scaffolds, as it governs the balance between rapid antimicrobial action and long-term therapeutic efficacy. Achieving this balance requires tuning both the material composition and the release mechanisms inherent to the scaffold architecture.

One common release pattern is the burst–plateau profile, as observed in plasma-activated PCL scaffolds. These materials exhibit a sharp initial release of Ag^+^ within the first 12 h, providing an effective response against acute bacterial contamination followed by a sustained, lower-rate release extending over two weeks. This dual-phase behavior ensures both immediate microbial suppression and continued protection during tissue healing [103].

In another approach, enzymatic degradation-driven release plays a central role in collagen-based systems. When AgNPs are generated in situ within the collagen matrix, the resulting scaffold demonstrates enhanced resistance to enzymatic breakdown. This property not only prolongs structural integrity, but also enables a more gradual and sustained release of silver ions over time, aligning with the slower progression of biofilm formation and chronic infection risks [101].

Lastly, diffusion-regulated ion release can be achieved by manipulating the scaffold’s internal architecture and composition. Materials incorporating tailored porosity or bioactive fillers, such as hydroxyapatite or graphene oxide, exhibit more controlled Ag^+^ diffusion profiles. These modifications create selective ion transport pathways, allowing for fine-tuned release rates that match therapeutic demands while minimizing cytotoxic effects [105].

Surface modification of polymer scaffolds plays an important role in optimizing nanoparticle binding, ensuring uniform dispersion, and enhancing cell–material interactions. These modifications not only improve the physicochemical compatibility between the polymer and AgNPs but also contribute to the scaffold’s biological performance.

Plasma activation is one such technique, commonly applied to PCL fibers to introduce reactive functional groups: primarily hydroxyl (–OH) and carboxyl (–COOH) onto the scaffold surface. These groups serve as effective anchoring points for the formation or immobilization of AgNPs, improving their stability and distribution across the matrix [103]. Alternatively, polydopamine coatings provide a multifunctional surface layer. Acting both as a mild reducing agent and an adhesion promoter, polydopamine facilitates localized nucleation of AgNPs while simultaneously enhancing scaffold hydrophilicity and wettability, key attributes for cell attachment and proliferation [102].

The addition of collagen coating further enhances the biological interface. As a natural extracellular matrix component, collagen offers excellent biocompatibility and provides specific binding sites for nanoparticle attachment. This dual functionality supports both tissue regeneration and antibacterial activity, making it particularly advantageous in wound healing or bone tissue engineering contexts [104]. Finally, the inclusion of bioactive fillers, such as calcium phosphate, graphene oxide, or nano-hydroxyapatite, introduces supplementary platforms for AgNPs functionalization. These materials not only reinforce the mechanical integrity of the scaffold but also provide a chemically active surface that can further enhance osteoconductivity, antimicrobial efficacy, or electrical responsiveness, depending on the application [105].

### 4.4. 3D/4D Printed Structures

Four-dimensional printing with AgNPs composites faces challenges including maintaining nanoparticle dispersion, avoiding release of spikes during processing, ensuring repeatable actuation, and relying on standardized fatigue/release testing protocols.

The emergence of 3D and 4D printing technologies has significantly advanced fabrication strategies for polymer-based AgNPs systems, enabling precise spatial control over scaffold architecture and functionalization. These technologies allow precise control over architecture, mechanical properties, and localized AgNPs distribution, thus expanding applications in wound healing, bone regeneration, and smart biomedical devices. Four-dimensional printing is an advanced form of 3D printing in which the printed object is designed to change its shape, properties, or function over time in response to external stimuli such as temperature, moisture, pH, light, and magnetic or electric fields.

In in situ approaches, silver precursors (e.g., AgNO_3_) are incorporated into polymer matrices prior to printing, followed by chemical or photochemical reduction post-deposition. For example, collagen scaffolds printed using extrusion-based bioprinting and exposed to UV light can induce the formation of AgNPs in the range of 10–28 nm directly within the matrix [101].

In contrast, ex situ strategies deposit pre-synthesized AgNPs onto the surface of printed scaffolds. This is often achieved by surface modification with adhesive layers such as polydopamine, enabling the immobilization of AgNPs and enhancing antimicrobial coverage across scaffold surfaces [106]. Both methods demonstrate unique advantages: in situ integration offers homogeneous nanoparticle distribution, while ex situ coating ensures surface-level potency with minimal Ag leaching.

Thermoplastic polymers such as PCL and PLGA acid maintain structural integrity post-printing due to their intrinsic melting–solidification behavior. In one study, AgNPs embedded in 3D-printed PLGA scaffolds resulted in robust architectures with antibacterial activity and mechanical stability suitable for bone tissue applications [107]. Alternatively, hydrogel-based systems, such as alginate, gelatin, or collagen, require ionic (e.g., Ca^2+^) or photochemical crosslinking for mechanical stability. When combined with AgNPs, these networks not only become structurally reinforced but also demonstrate enhanced antibacterial effects and delayed silver ion release [108].

AgNPs release from printed constructs depends on the matrix degradation rate, nanoparticle–polymer interaction, and environmental pH. For instance, PCL-based scaffolds allow for slow degradation over 3–6 weeks, facilitating sustained AgNPs and Ag^+^ release with prolonged antimicrobial effects once the scaffold is degrading [109]. On the other hand, hydrogel matrices can be tailored to release AgNPs in a burst-sustained profile, which is especially useful for wound dressing applications. Incorporating nanocellulose or mesoporous bioactive glass nanoparticles into hydrogels has also been shown to slow release kinetics and stabilize AgNPs against aggregation and oxidation [110].

Surface functionalization enhances the antifouling and antibacterial performance of printed scaffolds. Polydopamine coatings serve as reductive, bioadhesive intermediaries for AgNPs immobilization, significantly reducing microbial adhesion and biofilm formation [106].

Functionalized 3D-printed scaffolds have achieved significant decreases in *Staphylococcus aureus* and *Escherichia coli* colonization. Moreover, hybrid filler systems, such as Cu-Ag-doped mesoporous bioactive glass nanoparticles integrated into alginate/PVA scaffolds, simultaneously support angiogenesis, and provide long-term antimicrobial protection, demonstrating the potential of combinatorial surface engineering strategies [111].

### 4.5. Coatings and Sprays

Silver nanoparticle coatings and sprayable formulations are other variants which provide versatile, scalable, and surface-specific strategies for endowing substrates with antimicrobial and bioactive functions. These systems are ideal for post-fabrication functionalization of biomedical devices, textiles, consumer products, and packaging materials, offering flexibility in application and sustained efficacy.

Ex situ incorporation involves embedding pre-synthesized AgNPs into a polymeric or solvent-based matrix which is subsequently applied to surfaces via spraying, dip-coating, spin-coating, or brush-painting. For instance, AgNPs dispersed in PVA were successfully spray-coated onto textiles, producing durable antibacterial fabrics that retained >99.9% activity even after multiple laundering cycles [112].

In contrast, in situ generation entails applying silver salts to the target surface followed by chemical or photochemical reduction to form AgNPs directly on-site. Surfaces functionalized with dopamine or other reductive groups can catalyze silver ion reduction while anchoring nanoparticles tightly, increasing durability, and minimizing leaching [113].

Polymer-based networks have emerged as essential platforms for enhancing the stability and functionality of AgNPs-containing coatings. Both natural and synthetic polymers, such as chitosan, PVA, gelatin, and polyurethanes, provide a matrix that immobilizes silver nanoparticles while simultaneously improving wear resistance and mechanical durability [114]. These polymeric systems can undergo crosslinking through thermal, enzymatic, or photochemical methods, allowing for fine-tuned mechanical properties and regulated silver ion release. A notable example involves spray-deposited hydrogels composed of gelatin methacrylate integrated with AgNPs. Upon in situ photo-crosslinking, the hydrogels formed flexible, adherent films that not only conformed to irregular wound surfaces but also maintained robust antibacterial activity and mechanical resilience under shear stress [115].

Maintaining the stability of sprayable AgNPs formulations is essential to ensure a balance between antimicrobial efficacy and cytocompatibility. An uncontrolled burst release of Ag^+^ may lead to acute cytotoxic effects or transient antimicrobial action, whereas insufficient ion availability can render the formulation therapeutically ineffective. To address these challenges, several stabilization strategies have been developed to regulate silver ion release kinetics under physiological conditions.

One widely employed approach involves polymer entrapment, wherein AgNPs are embedded within hydrophilic matrices such as hydrogels or PVA films. These systems provide a hydrated environment that acts as a diffusion barrier, moderating ion release over time [116]. Alternatively, layer-by-layer assembly techniques utilize the sequential deposition of oppositely charged polymers and AgNPs to fabricate nanostructured coatings. These multilayered architectures enable precise control over nanoparticle loading and release profiles through layer thickness and composition tuning [117].

Another effective stabilization method is core–shell encapsulation, in which AgNPs are coated with protective shells such as silica or PEG. These coatings enhance oxidative stability and prolong the functional lifespan of the nanoparticles under biological conditions by mitigating premature ion leaching [118].

In addition to passive stabilization, stimuli-responsive release systems have emerged as promising tools for controlled delivery. For example, pH-sensitive or thermoresponsive formulations can exploit the acidic microenvironment of infected or wounded tissues to trigger localized Ag^+^ release, thereby enhancing targeted antimicrobial activity while limiting systemic exposure [119].

Achieving durable adhesion of AgNPs-based coatings to diverse substrate materials remains a critical challenge, particularly in biomedical and high-stress applications. To address this, various surface functionalization techniques have been developed to improve the interfacial compatibility between AgNPs and underlying substrates. One widely employed approach is plasma treatment, which is especially effective for low-surface-energy polymers such as PE. Exposure to plasma generates reactive functional groups (e.g., hydroxyl, carboxyl) on the polymer surface, thereby increasing surface energy and enhancing the interaction with AgNPs or their carrier matrices [120]. Another versatile strategy involves polydopamine priming. Inspired by mussel adhesive proteins, polydopamine forms a conformal layer rich in catechol and amine groups that can chelate silver ions and facilitate robust nanoparticle anchoring. This bioinspired coating not only promotes adhesion but also contributes to improved surface wettability and biocompatibility [113].

For inorganic substrates, such as glass or ceramics, silane coupling agents are commonly employed. These bifunctional molecules form covalent bonds with hydroxylated surfaces on one end, while the other end can interact with AgNPs-containing coatings through chemical or physical means. This technique provides a strong and stable interface, particularly useful in environments requiring long-term mechanical integrity [121].

These surface modification strategies are particularly crucial in applications subjected to mechanical stress or moisture exposure, including surgical instruments, implantable devices, and wound-contact materials, where reliable coating adhesion is essential for sustained antimicrobial function and device performance.

## 5. Applications for Polymeric Systems Containing AgNPs

### 5.1. Biomedical Applications

#### 5.1.1. Antibacterial Wound Dressings

Wound dressing is one of the main applications of polymers matrices, keeping in mind the biomedical purpose. Wound dressings are essential in wound treatment, especially if the area of the affected organ is large. Skin, the largest organ in the human body, can be altered due to various factors and stimuli. Furthermore, skin is permanently involved in metabolic changes from our body, so is it critical to ensure stability and normal physiological functions all the time [122]. Extensive cutaneous defects can precipitate systemic pathophysiological responses, including hypermetabolism, excessive transdermal loss of fluids and plasma proteins, immune dysregulation, functional impairment, and, in severe instances, mortality [123].

**Figure 6:**
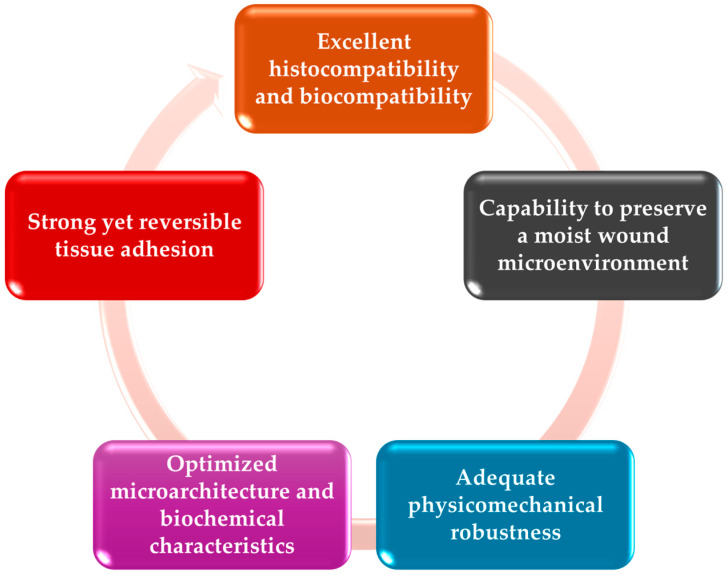
Main characteristics of wound dressings regarding biocompatibility, swelling properties, mechanical and architectural features, and tissue tolerance.

Nowadays, several types of wound dressing are available, designed to offer temporary support, to protect the infected wound, and to assure a prevention of microbial infection. The polymeric basis of the wound dressing is the perfect matrix for healing, while regeneration or antibacterial effect is assured by the drugs/nanoparticles incorporated. Various wound dressing types have been fabricated lately. Some examples are hydrocolloid dressings, hydrogels, foam dressings, films, and membranes [124]. Regardless of the type of wound dressing, the polymeric material fabricated should follow some specific characteristics described briefly in Figure 6 [125].

**Figure 7:**
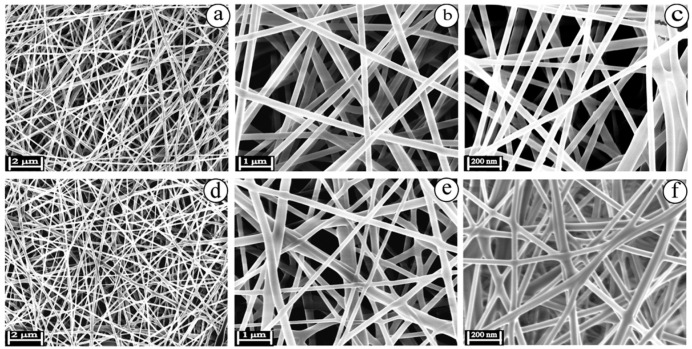
SEM for electrospun AgNPs nanofibers: (**a**–**c**) for Eugenol-AgNPs nanofibers at various magnifications, and (**d**–**f**) for Silver bandaid suspensions-AgNPs nanofibers at various magnifications. Reprinted from an open-source article [126].

Several studies have been reported for wound dressing based on AgNPs–polymer composite. Sethuram et al. [126] performed a comparison between eugenol-AgNPs and silver bandaid suspensions for wound infections treatment. The samples were fabricated using electrospinning and they were evaluated optically and physiochemically. Also, the antibacterial effect was confirmed using MICand in vitro ion release. SEM results, presented in Figure 7, confirmed the nanofibrous structure for the material tested, showing that eugenol components have a homogenous distribution while being conjugated with AgNPs [126]. Biological tests and cytotoxicity activity proved a better activity for eugenol-AgNPs, showing less toxicity for the cells tested.

Another example is the cellulose–HA/AgNPs injectable hydrogel developed by Long et al. which achieved 100% closure of burn wounds in mice within 14 days, demonstrating both rapid healing and infection control [127]. AgNPs also showed results in healing chronic wounds. Green-synthesized polysaccharide hydrogels loaded with AgNPs showed significant bacterial suppression and accelerated closure in diabetic mice models within 14 days, as reported by [90]. Alipour et al. [128] reported the fabrication of a three-layered antibacterial hydrogel with green-synthesized AgNPs incorporated for infection control and wound healing. The mixture of natural polymers, such as chitosan and hyaluronic acid, with synthetic polymers, PVA, and lidocaine as anesthetic agent showed an improved stability leading to a multifunctional device for wound healing [128].

#### 5.1.2. Drug Delivery and Controlled Release Systems

Polymer matrices-based drug delivery systems have emerged as novel tools in controlled and sustained therapeutic agent release, particularly for localized applications. When integrated with AgNPs, these systems offer not only physical support for drug loading and transport, but also inherent antimicrobial properties, making them highly effective against resistant pathogens. The synergy between AgNPs and encapsulated drugs enhances therapeutic efficacy while potentially reducing the required dosage and limiting side effects [129]. Embedding AgNPs in hydrophilic, cross-linked polymeric networks allows for site-specific, stimuli-responsive drug release, which is critical in treating infections in wounds, surgical sites, or internal tissues. For instance, chitosan-based hydrogels loaded with AgNPs and curcumin/ciprofloxacin demonstrated controlled release and improved antibacterial performance against *Escherichia coli* and *Staphylococcus aureus* due to the synergistic effects of both agents [130]. The hydrogel structure ensures sustained release, while the AgNPs inhibit early-stage bacterial colonization and biofilm formation.

Additionally, PVA/AgNPs hydrogels have been engineered for wound infection management, releasing both silver ions and encapsulated antimicrobial drugs under physiological conditions. One such study utilized PVA-AgNPs hydrogels loaded with curcumin, where AgNPs enhanced the antibacterial range, and curcumin provided anti-inflammatory effects, supporting both disinfection and tissue regeneration [131]. This combination was especially potent against multi-drug-resistant bacterial strains.

Moreover, stimuli-responsive hydrogels, particularly pH or temperature-sensitive systems can be designed to release drugs preferentially at infection sites where acidic pH or elevated temperatures are common due to inflammation. A recent work demonstrated the use of thermoresponsive hydrogel–AgNPs composites for transdermal drug delivery. The system remained stable at room temperature but released both AgNPs and the encapsulated antibiotic upon reaching skin temperature (37 °C), enhancing treatment efficiency while minimizing systemic exposure [132].

In situ-forming injectable hydrogels with AgNPs also offer promising drug delivery strategies for internal infections. These systems can be applied in minimally invasive procedures and solidify at the target site, forming a localized reservoir that releases both the AgNPs and therapeutic agents over time, which is ideal for applications like periodontal disease, osteomyelitis, or infected implants [133].

Despite their advantages, the release kinetics of AgNPs and drugs must be carefully controlled to avoid cytotoxicity and ensure biocompatibility. Surface modification of AgNPs, polymer blending, and crosslinking density adjustments are common strategies to fine-tune these properties [134].

#### 5.1.3. Tissue Regeneration and Scaffolding

Tissue engineering, an important field of nanotechnology, has the aim to create innovative materials for disrupted human body functions. In tissue engineering, the materials should possess high biocompatibility and should simulate in high percentage the extracellular matrix of the implanted area. Considering these properties, the possibility of implant or scaffold rejection is minimal, while the healing is assured by the cells from the surrounding tissues. Scaffolds can be fabricated for soft tissue engineering or hard tissue engineering, if bone restauration is needed. Classical synthesis methods are well known in the literature; however, the hot topic of tissue engineering remains, at the moment, the bioprinting approach.

The advent of 3D and 4D printing technologies has opened new frontiers in designing AgNPs-loaded polymeric systems with unprecedented spatial precision and dynamic functionality. Three-dimensional printing allows for layer-by-layer fabrication of complex architectures, enabling precise distribution of silver nanoparticles within defined regions of the structure. This approach supports patient-specific implants, smart wound dressings, and compartmentalized drug release systems [56]. Four-dimensional printing further extends this capability by incorporating stimuli-responsive materials, enabling structures that change shape or function over time in response to external triggers—such as temperature, moisture, or light [135]. These technologies not only improve functional customization but also enhance reproducibility and scalability in advanced manufacturing settings.

Together, these polymeric platforms and fabrication techniques provide a flexible toolbox for addressing the multifaceted challenges of antimicrobial delivery and material performance in both clinical and commercial environments. Their integration with AgNPs continues to push the boundaries of smart, multifunctional materials for next-generation biomedical devices and food safety systems.

The versatility of 3D/4D-printed AgNPs–polymer systems enable a broad spectrum of biomedical applications, which are summarized in Table 3.

Four-dimensional printing, though still emerging in the AgNPs–polymer domain, offers transformative potential. Shape-memory and stimuli-responsive hydrogels (responsive to pH, temperature, or moisture) have been developed for smart wound dressings and implants that can adapt in vivo conditions [136].

### 5.2. Food Packaging Applications

**Figure 8:**
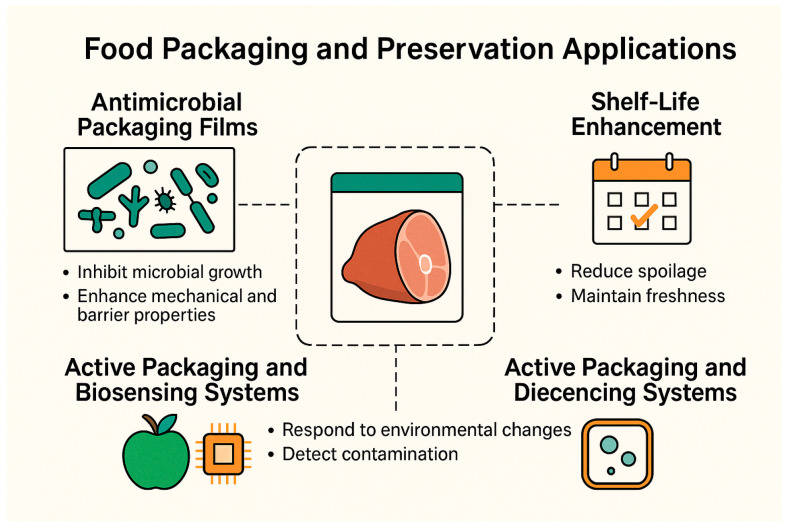
Food packaging approaches regarding AgNPs–polymer systems.

This chapter delves into the implementation of AgNPs–polymer platforms in food packaging, highlighting their roles in antimicrobial protection, shelf-life extension, and emerging active/biosensing functionalities. Figure 8 illustrates the main functional areas: antimicrobial packaging films that inhibit microbial growth at the food–package interface, shelf-life enhancement through oxidative and microbial spoilage reduction, and active packaging systems integrated with biosensors for real-time freshness monitoring.

#### 5.2.1. Antimicrobial Packaging Films

AgNPs-integrated packaging films are engineered to actively inhibit microbial proliferation at the food–package interface. Their antimicrobial functionality is primarily mediated through two synergistic mechanisms: the sustained release of silver ions and direct interactions between nanoparticles and microbial cells. These mechanisms result in potent antibacterial and antifungal effects, while the inclusion of AgNPs also contributes to improved mechanical robustness and enhanced barrier properties, particularly against water vapor and gas transmission [137].

A range of biodegradable and food-safe polymers serve as matrices for AgNPs incorporation. Materials such as cellulose derivatives, chitosan, starch, alginate, pectin, and PVA are commonly employed due to their film-forming ability and environmental compatibility. Two principal strategies are used to incorporate AgNPs into these matrices. The in situ synthesis approach involves the introduction of silver salts directly into the polymer solution before casting. During the drying phase, or under mild heating, reduction reactions take place, generating nanoparticles that are uniformly distributed throughout the film. This method offers strong matrix integration and reduced aggregation. Alternatively, ex situ methods rely on pre-synthesized AgNPs, which are either mixed into the polymer blend or applied to film surfaces post-casting. This approach enables precise control over nanoparticle size and morphology, although achieving homogenous dispersion can present challenges. Both incorporation methods have demonstrated notable antimicrobial efficacy. For example, carboxymethylcellulose-based films containing AgNPs have shown strong inhibitory effects against *Escherichia coli*, even at extremely low silver concentrations (\~0.1 µg/cm^3^) [138]. In another study, pectin/gelatin films embedded with AgNPs and curcumin achieved nearly complete suppression of *Escherichia coli* and *Staphylococcus aureus*. These films also displayed improved water vapor barrier characteristics and mechanical properties, and acted as pH-sensitive indicators through distinct color changes (from yellow to deep red), allowing for real-time freshness monitoring in shrimp packaging applications [139].

Beyond microbial inhibition, AgNPs also enhance the physical characteristics of packaging films. Their inclusion reduces water vapor permeability and oxygen transmission rates by filling microvoids in the polymer matrix and increasing film density, both of which are critical for the preservation of moisture-sensitive food products. Mechanical strength and thermal stability are also improved, as demonstrated in glutaraldehyde-crosslinked PVA/sodium caseinate films containing AgNPs, which exhibited increased tensile strength and reduced swelling behavior [140]. In practical applications, such films have been used to package perishable produce such as asparagus and fruits, where they effectively maintained texture and delayed microbial spoilage during refrigerated storage [137]. Furthermore, conductive polymers such as polyaniline and PEDOT, when integrated with AgNPs, endow the packaging with volatile gas sensing capabilities. These materials can detect gases like ammonia or acetone, enabling simultaneous antimicrobial protection and spoilage monitoring [141].

#### 5.2.2. Shelf-Life Extension Through AgNP–Polymer Packaging

Polymer films embedded with AgNPs have emerged as a versatile platform for extending the shelf life of a wide array of food products. Their multifunctional character, encompassing antimicrobial activity, antioxidant potential, and improved barrier properties, makes them highly effective in mitigating spoilage, preserving organoleptic quality, and retaining the nutritional integrity of packaged foods. By addressing multiple degradation pathways simultaneously, AgNPs-based packaging provides a strategic advantage over conventional materials in food preservation.

The mechanisms underpinning the shelf-life extension offered by AgNPs–polymer composites are multifaceted; however, they are similar to the previously presented mechanisms of AgNPs. A primary factor is microbial suppression, achieved through the gradual release of AgNPs and Ag^+^ ions from the packaging in time. Since the bacteria are usually found on the surface or in the product, the release of both AgNPs and Ag^+^ in time can increase the stability of the product. In addition to antimicrobial effects, AgNPs exhibit antioxidant capabilities [142], particularly in oil-rich food systems. Their ability to scavenge free radicals and interact with oxidizing agents helps to limit lipid peroxidation, reducing rancidity and preserving flavor stability [143]. Moreover, the incorporation of AgNPs into polymer matrices contributes to a denser film microstructure. This densification diminishes the permeability of the films to oxygen and water vapor, two key accelerants of physical and biochemical spoilage. When these mechanisms act in concert, the result is a marked delay in the onset of quality degradation, enabling the retention of freshness, texture, and flavor for extended durations.

The efficacy of AgNPs-based packaging has been demonstrated across a variety of food matrices. For instance, in long-term storage studies involving shelled nuts such as almonds, hazelnuts, pistachios, and walnuts, films containing 1–3 wt% AgNPs significantly reduced microbial and mold growth over a 24-month period. In particular, pistachios packaged with high AgNPs-loading films exhibited a notable increase in shelf life, from approximately 13 months to 20 months, while also preventing aflatoxin formation. Importantly, these preservative effects were achieved without compromising sensory quality [144].

In high-moisture, lipid-rich food systems such as oil cakes, PE films doped with 3–5 wt% AgNPs and TiO_2_ nanoparticles were shown to suppress microbial spoilage effectively during ambient storage (~25 °C). These nanocomposite films not only exhibited enhanced antimicrobial action, but also improved moisture barrier function, enabling the extension of product shelf life from 30 to approximately 37 days compared to non-nanoparticle controls [145]. Similarly, cellulose–chitosan films incorporating AgNPs/TiO_2_ composites achieved over 79% inhibition of mold and yeast growth, successfully maintaining the quality of fresh produce for up to six months under refrigerated conditions [146]. In another study, chitosan films enriched with both essential oils and AgNPs were used to pack strawberries. The resulting system effectively slowed spoilage at 4 °C, preserving both visual appeal and textural integrity over several weeks of storage [147]. These case studies highlight the critical design variables that govern preservation efficacy in AgNP-based packaging systems. The loading level of AgNPs plays a pivotal role; while higher concentrations are often associated with enhanced microbial suppression and shelf-life gains, they also introduce considerations related to cost, regulatory compliance, and potential toxicity. Lower concentrations can still deliver meaningful preservation benefits, particularly when combined with synergistic additives.

AgNPs-based packaging materials have demonstrated considerable potential in extending shelf life and enhancing food safety; however, their widespread implementation remains constrained by several critical challenges. Among these are regulatory concerns related to silver migration and overall nanoparticle content. To ensure consumer safety, packaging systems must be engineered to limit the release of Ag^+^ to within legally permissible thresholds. This requires precise control over nanoparticle loading, dispersion, and matrix compatibility to prevent unintended leaching during storage and use. Another key consideration is the potential sensory impact of AgNPs-containing films. Elevated concentrations of AgNPs, though effective in microbial suppression, have occasionally been associated with alterations in the flavor, aroma, or visual appearance of food products. Consequently, rigorous sensory evaluation is necessary to confirm that packaging materials do not compromise the organoleptic qualities of the food they are intended to protect.

#### 5.2.3. Active Packaging and Biosensing Systems

In recent years, the development of AgNPs–polymer films have progressed beyond passive food protection, moving toward multifunctional systems capable of actively monitoring food freshness. These advanced packaging materials not only inhibit microbial growth, but also incorporate biosensing functionalities, often based on colorimetric or gas-responsive mechanisms, that signal spoilage in real time. Such smart systems enable freshness assessment visually or electronically without the need to breach the packaging, offering practical benefits across the supply chain.

One prevalent sensing strategy involves the detection of spoilage gases, particularly hydrogen sulfide, which serves as a reliable marker of meat degradation. A notable example is a gellan gum–AgNPs bionanocomposite that detects hydrogen sulfide released during meat spoilage. The interaction between AgNPs and hydrogen sulfide induces the formation of Ag_2_S, producing a visible color shift from yellow to colorless. This system demonstrates high selectivity, a detection limit of approximately 0.81 µM, and enables nondestructive, in situ monitoring, representing a robust and cost-effective approach to spoilage detection [148]. The functional performance of these biosensing systems is tightly linked to their material design and composite architecture. Responsive dyes or functional nanostructures are embedded alongside AgNPs within food-safe polymer matrices to deliver dual antimicrobial and sensing capabilities. One example includes curcumin-loaded chitosan/polyvinyl alcohol films, which display a gradual color shift from yellow to red in response to shrimp spoilage. These films not only signal freshness but also achieve 2–3 log reductions in *Escherichia coli* and *Staphylococcus aureus*, while maintaining mechanical strength and barrier properties [149]. These hybrid composites illustrate the synergistic integration of sensing and microbial suppression into a single, multifunctional film with the main advantage of real-time monitoring. Most are capable of generating visible signals within a few hours and remain stable under both refrigerated and ambient conditions, making them suitable for broad deployment in food packaging.

## 6. Challenges and Limitations

**Figure 9:**
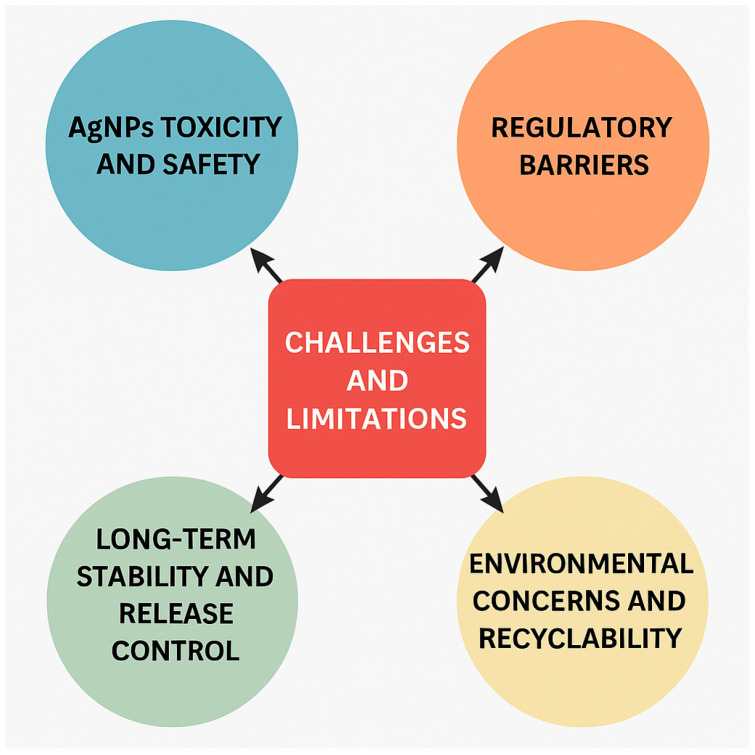
Challenges and limitations of AgNPs in biomedical and food applications.

The challenges regarding AgNPs, presented schematically in Figure 9, must be discussed since they interact with other biological structures or structures from ecosystems. Lately, regulation regarding AgNPs is focused on their use in the food industry, since this domain is more permissive compared to biomedical application. However, regardless of the final application, the possible accumulation of AgNPs must be avoided.

### 6.1. AgNPs Toxicity and Safety

A number of key toxicological concerns have been identified in relation to AgNPs, particularly regarding their interactions with human biological systems. The significant antimicrobial activity of AgNPs derives largely from Ag^+^ release and the generation of ROS, DNA damage, and apoptosis or necrosis; however, these same mechanisms pose risks to human health. As reviewed by [150], cytotoxicity depends on nanoparticle size, surface charge, concentration, route of exposure, and functionalization method. In vitro and in vivo studies demonstrate that AgNPs can induce oxidative stress, inflammation, mitochondrial damage, genotoxicity, and apoptosis in various cell types, including hepatocytes, immune cells, reproductive cells, and neural tissues [151]. Zhang et al. [151] summarized mechanistic pathways across organ systems, noting that inhalation, oral, and dermal exposures can lead to dose-dependent damage to the lungs, liver, reproductive tract, and gut microbiota [151].

The antibacterial effect of AgNPs is a promising strategy for micro-organisms’ inhibition; however, many in vitro and in vivo studies revealed the possible toxicological behavior of AgNPs on cells and bacteria. These types of experiments involve the exposure of AgNPs to cultures or organs in different doses, thus evaluating the final reactions. Interactions are mainly guided by the concentrations and doses of AgNPs; therefore, a dose-dependent is analyzed using cytotoxic assays [150] to establish the toxic effects on human health. In the case of nanoparticles, the reactions analyzed are quite different than the classical cellular toxicity investigation [152]. For AgNPs toxicological considerations, aggregation at specific areas is investigated using techniques such as colorimetric tests or biological analysis. Genetic analysis and molecular biology kits are also methods to investigate the possible cell uptake of AgNPs high doses [150].

The influence of AgNPs can appear at very early stages of development as it is also proved in a recent research paper presented by Il Kim et al. [153]. The study is evaluating the side effects of AgNPs on an embryo culture model from rats in order to observe the embryonic growth and development while exposed to different doses of AgNPs. Using TUNEL technique and immunohistochemistry as main methods for detection, the results revealed that a 15 μg/mL dose of AgNPs will induce significant medical issues such as retardation in growth, morphological abnormalities, or even the absence of some organelles. Confirming the dose-dependent effect, at 1.67 μg/mL AgNPs, no harmful effects were observed for the direct exposure in the embryonic growth [153]. Similar observations were identified in a complex research study performed by Quevedo et al. [154] in which the toxicity of AgNPs on embryonic zebrafish cells (ZF4) was addressed. Many parameters, such as various AgNPs concentrations, and different sizes and forms of the silver particles, were analyzed to reveal the pathways and mechanisms for cells deaths. Using a range of AgNPs concentrations between 5 and 60 μg/mL and three different sizes, 10, 30, and 100 nm, many assays were tested to determine the effective concentration of AgNPs. Lactate dehydrogenase activity assay showed a decrease in cell viability for bigger particles, the ones of 30 and 100 nm at concentrations higher than 20 μg/mL in the first hours of exposure. In time, the viability decreased for all three sizes; however, the toxicity for the 10 nm AgNPs was indicated to be slightly smaller. The relationship between the other tests of the study showed that the 100 nm size induces peroxidation and autophagy reactions at their lowest concentrations, while higher concentrations decrease the autophagy level. The authors also proved the influence of NPs size and doses over mitochondrial permeabilization through mitochondrial pathways, reinforcing the assumptions of the scientific world according to which AgNPs act at the mitochondrial level, causing cellular necrosis [154]. The impact of the size regarding the toxicological considerations is also important for the antibacterial effect in bacteria culture since many reports have shown an impact on bacteria inhibition. A stronger antimicrobial effect is associated with the decrease in AgNPs dimension due to a faster and easier uptake of the particles, as reported by various scientific papers [155,156]. Another in vitro study showed that, in addition to the size and the dose-dependent property, the toxicity of AgNPs also depends on the media to which they are exposed [157]. Kim et al. [157] tested uncoated and coated AgNPs (polymer and citrate) with two different sizes, 20 and 110 nm, on zebrafish embryos. They tested three different media, CaCl_2_, pure water, and embryo medium, to identify the conditions in which AgNPs express their strongest toxicity. The results of the tests confirm the other studies that show that smaller AgNPs, the ones of 20 nm, are more toxic compared to the others, while the polymer-coated AgNPs are also more toxic than the ones with a citrate attachment. The results proved that by using CaCl_2_ and pure water, the tendency of AgNPs to agglomerate is lower, while in embryo medium, the particles showed a higher toxicity, having a tendency to agglomerate in the culture [157]. The association of AgNPs with a polymer coating on their surface was investigated also by Lee et al. [158]. Their toxicological study was also related to a surface coating-dependent behavior of AgNPs using zebrafish embryos. Coated AgNPs-PEG and AgNPs-citrate were evaluated using various ionic environments and the results proved different dissolution and aggregation, and, therefore, also differences in the toxicity of the embryos tested. The results confirm a correlation in the AgNPs dissolution and their toxicity, showing that AgNPs-PEG are less toxic in ionic environment and more stable over time due to the protection of PEG [158]. All these studies have proved that AgNPs toxicity depends on many parameters. The main factors remain their concentration, the dose-dependent property, and their sizes; so, to confirm the biocompatibility required for this type of materials, different biological analysis should be performed over time.

Compared to in vitro studies, the AgNPs toxicity in humans is much less studied in the scientific community, especially due to the ethical considerations. However, existing in vivo studies on animals exposed various pathways for AgNPs, such as inhalation, ingestion, or direct application on the skin [150]. Also, for this type of cytotoxicity, the dose, concentration, size, and shape remain the main factors to be considered.

Occupational exposure limits have been established. For instance, National Institute for Occupational Safety and Health (NIOSH) recommends a respirable 8 h Time-Weighted Average (TWA) of 0.9 µg/m^3^ for AgNPs with sizes < 100 nm (an 8 h TWA is the average exposure to a hazardous substance over an 8 h workday, assuming a 40 h workweek). The ionic form is considered the ultimate toxic species, with extracellular release driving cellular damage [159]. The absorption, distribution, metabolism, and excretion of AgNPs remain incompletely characterized, complicating prediction of chronic or cumulative effects [151]. Moreover, long term and multigenerational studies are largely absent, highlighting the need for more comprehensive assessments to support safe and responsible use of AgNPs in consumer and industrial applications. To our knowledge, no single universal “safe dose” exists; rather, effective safety depends on specific release rates under realistic conditions. Future studies will clarify this aspect.

In addition to the concern for acute exposure, the potential for chronic exposure effects, bioaccumulation, and long-term safety concerns are crucial aspects of responsible nanomaterial design. The literature indicates a significant knowledge gap in this area, underscoring the need for a more comprehensive view of nanotoxicity [160].

Chronic, low-concentration exposure is a primary concern. Unlike acute effects, which may be immediate and dramatic, the subtle, long-term impact of continuous exposure to AgNPs is still not fully understood. Studies have shown that even low concentrations of certain heavy metals can affect biological systems and immune responses without obvious clinical manifestations, suggesting that chronic exposure might have adverse effects on overall health [161]. This concern is magnified by the potential for bioaccumulation, where AgNPs, due to their small size, can traverse biological barriers and accumulate in tissues over time [162]. For instance, fungi are known to have a capacity to bioaccumulate metals, highlighting a potential pathway for nanoparticles to enter and persist in biological systems [163]. This accumulation raises a significant question about the long-term safety of these materials in applications where they may be internalized or have prolonged contact with the body.

Furthermore, we must address the lack of standardized toxicity testing protocols for AgNPs–polymer composites, which is a major hurdle in evaluating long-term safety [164,165]. The absence of universally accepted methods for in vitro and in vivo testing leads to a high degree of variability and a lack of comparability between studies. This makes it difficult to establish robust safety profiles for new materials. This problem is compounded by the unique physicochemical properties of nanoparticles, such as their small size and high surface area, which can interfere with standard testing methods like optical density measurements [165]. To overcome this, there is a clear call in the literature for the development of validated, standardized assays that can accurately assess nanotoxicity, allowing for better correlations between in vitro results and in vivo outcomes [165].

### 6.2. Regulatory Barriers

The regulatory landscape presents formidable challenges for AgNPs–polymer systems, especially regarding their use in food contact materials. While AgNPs offer significant antimicrobial advantages, their nanoscale complexity triggers strict regulatory scrutiny, which varies considerably across jurisdictions.

In the European Union, Regulation (EC) No. 1935/2004 requires that any substance used in food contact materials must be demonstrated not to compromise human health or alter food in unacceptable ways. This extends to nanomaterials—even those derived from previously approved bulk substances. Under Regulation (EU) No. 10/2011, materials are evaluated individually, and silver in nanoform is not currently listed in Annex I, meaning nanosilver is not authorized for general use in the EU without undergoing a formal approval procedure [166]. In response, the European Food Safety Authority (EFSA) published detailed guidance in 2021 outlining the specific data needed for nanomaterial assessments, such as accurate characterization of particle size distributions, solubility, and migration behavior, as well as standardized toxicological studies [167]. EFSA’s authoritative safety assessment, also released in 2021, concluded that plastics containing up to 0.025% (*w*/*w*) nanosilver release no more than 6 µg/kg of silver into food, well within the accepted 50 µg/kg limit and below the acceptable daily intake of 0.9 µg/kg body weight/day [166]. However, EFSA emphasized that applications must still include nanoscale-specific testing to fulfill safety requirements. EFSA revisited silver-based food additives (E 174) in April 2025 and reaffirmed concerns claiming that data on nanoparticle characteristics, long-term toxicity, and genotoxicity remain insufficient, preventing conclusive safety approval of silver nanoparticles in food contact materials [168].

In the United States, AgNPs-based materials are regulated via the Food and Drug Administration (FDA) process or through GRAS (Generally Recognized as Safe) determinations. As of yet, no AgNPs packaging material has received federal approval. Regarding the biomaterials, these evaluations must address both chronic and sub-chronic exposures, as well as genotoxic potential, to meet the stringent standards set by regulatory bodies. Even so, applicants must demonstrate rigorous nanotoxicological and migration testing. Because of their biocidal function, AgNPs products may also fall under Environmental Protection Agency (EPA) or National Institute for Occupational Safety and Health (NIOSH) regulation, especially where antimicrobial claims are involved [169].

Globally, the regulatory environment is fragmented. Canada lacks explicit nanoparticle migration thresholds, Brazil excludes AgNPs from approved plastic additives, and several countries, such as India, China, and Australia, either lack dedicated nanomaterial provisions or apply non-uniform guidance [170]. The regulatory approval of AgNPs-based food packaging remains a complex and fragmented process, presenting several key challenges to commercialization. In many jurisdictions, including the European Union, AgNPs are not universally authorized for use in food-contact materials. Instead, each application must undergo a case-by-case evaluation, which slows the approval process and adds considerable regulatory burden. Adding to this complexity is the lack of standardization in testing methodologies. Variations in the choice of food simulants, exposure conditions, and analytical detection limits across studies can hinder reproducibility and make cross-comparison of results difficult. Such inconsistencies complicate the safety assessment process and may lead to divergent regulatory outcomes. Complicating matters further, a 2019 systematic review of 26 migration studies reported substantial inconsistencies in test procedures. Only two studies claimed no silver migration, but methodological flaws in control conditions and analytical methods were common, limiting inter-study comparability and reducing confidence in findings [171]. Table 4 illustrates the regulatory status of AgNPs in food contact materials across key markets.

### 6.3. Recommendations for Standardizing Characterization and Testing

The key to resolving these inconsistencies lies in a multi-faceted approach, beginning with the standardization of characterization methods to ensure the fundamental properties of the nanoparticles are consistently measured [13]. The performance of AgNPs is inextricably linked to their size, shape, and surface chemistry. Therefore, all studies should utilize a core set of techniques, including Transmission Electron Microscopy (TEM) and Scanning Electron Microscopy (SEM) for precise morphological and size-dependent analysis. UV-VIS spectroscopy is also essential for confirming the presence of the characteristic SPR band, which provides a simple yet effective indicator of particle formation and size. Additionally, techniques like X-ray Diffraction (XRD) and Fourier-transform infrared (FT-IR) spectroscopy are crucial for confirming crystal structure and polymer–nanoparticle interactions, respectively. By establishing these methods as a baseline, we can ensure that the fundamental building blocks of the material are understood and reported uniformly. Regarding the antimicrobial standardization testing protocol, a minimum set of standardized protocols, such as the disk diffusion method and the determination of the MIC, should be adopted. It is also imperative that researchers report all experimental conditions, including nanoparticle concentration, media pH, and temperature, to eliminate potential confounding factors and improve the reproducibility of the results.

Finally, the standardization of release kinetics evaluation is critical for both scientific and regulatory purposes [172]. The release of Ag^+^ is a dynamic process influenced by numerous factors. Future studies should standardize the method for measuring Ag^+^ release, for instance, by using Inductively Coupled Plasma Mass Spectrometry (ICP-MS) [173] in various simulated environments. The goal is not just to measure release but to understand its mechanism. As we noted, release curves often follow non-Fickian models, suggesting that both diffusion and polymer relaxation are at play. Consequently, we advocate for the modeling of release kinetics, which provides deeper mechanistic insight. Reporting the total silver content, the percentage of released silver over time, and the specific test conditions will be vital for enabling better comparisons of migration data across different materials, ultimately ensuring that these materials meet safety and regulatory standards.

### 6.4. Long-Term Stability and Release Control

Ensuring long-term stability and controlled release of AgNPs in polymer systems is essential for the safe and effective application of AgNPs–polymer biomaterials. Extension of shelf life, microbial protection, and compliance with safety regulations all depend on how these materials behave over time under realistic conditions.

Long-term integrity of AgNPs within polymer matrices is influenced by physicochemical interactions, environmental factors, and film composition. Nanoparticles’ migration and ion release can occur through mechanisms such as surface desorption, oxidation, polymer degradation, or mechanical wear [174]. The exposure to various chemicals, especially acidic ones, significantly accelerates Ag^+^ release. One study showed that under 70 °C in 3% acetic acid, films without stabilizing additives released about 1.3% total silver over six days, while those with antioxidants and light stabilizers released just ~0.15%, demonstrating that formulation plays a key role in stabilizing AgNPs [175]. Independent release kinetics studies with starch-based films containing AgNPs revealed complex mass-transfer profiles. Films produced via in situ synthesis exhibited higher release (≈0.141 mg Ag/dm^2^ in acetic acid) compared to ex situ AgNPs films, though still well below legal migration limits in the EU (10 mg/dm^2^) and MERCOSUR (8 mg/dm^2^) [176]. Release curves followed non-Fickian models, indicating that diffusion coupled with polymer relaxation influences migration behavior. Controlled release of active components, like thymol in AgNPs/PLA films, often follows non-Fickian kinetics, impacted by the composite structure of polymer and nanoparticle dispersion. In one study, films with 1 wt% AgNPs and thymol exhibited controlled thymol release while AgNPs release remained minimal under environmental simulants, achieving dual antimicrobial and antioxidant protection [69].

The release behavior and long-term stability of AgNPs within polymer-based systems are critically influenced by the design of the polymer matrix, the choice of surface stabilizers, and the processing conditions employed during fabrication. Surface functionalization plays a key role in modulating nanoparticle stability. Stabilizing agents such as polyvinylpyrrolidone, PEG, thiolated PEG, and protective coatings, ranging from silica and gold to lipid membranes, have been shown to significantly enhance resistance to oxidative degradation, dissolution, and leaching. These modifications contribute to prolonging the functional lifespan of AgNPs in active systems [69]. Among these strategies, lipid-coated AgNPs have demonstrated particular promise. Compared to uncoated or citrate-capped nanoparticles, these hybrid systems exhibit reduced oxidation rates and a more controlled, less toxic release profile under aqueous conditions, which are commonly encountered in food-contact environments. This behavior suggests improved compatibility and safety in applications where moisture exposure could otherwise accelerate Ag^+^ ion dissolution [69]. This underscores the need for durability-focused designs: the outer layers of packaging or coating must remain intact throughout intended use to prevent unintended exposure or loss of efficacy.

To conclude this subsection, the most effective approaches in achieving long-term stability and release control are steric/electrostatic stabilization [177], covalent anchoring to polymer matrices, in situ nucleation with strong capping groups [178], encapsulation in microphases or core–shells [179], and validation under accelerated aging protocols.

### 6.5. Environmental Concerns and Recyclability

The incorporation of AgNPs in polymer matrices introduces environmental complexities, ranging from lifecycle-level resource burdens to end-of-life ecosystem impacts and recyclability hurdles. Careful design and responsible deployment are essential to reconcile antimicrobial benefits with ecological sustainability.

Life cycle assessments consistently identify AgNPs synthesis, including silver extraction and nanoparticle fabrication, as the dominant contributor to the environmental burden, often exceeding downstream impacts by an order of magnitude. Reference [180] reports on conducting a cradle-to-gate life cycle assessments across seven AgNPs synthesis routes, finding that electricity-intensive processes such as flame spray pyrolysis and magnetron sputtering yielded the highest impacts across global warming, acidification, human health, and ecotoxicity indicators. Even bio-based routes using starch as a reducing agent showed unexpectedly high ecotoxicity when scaled for performance efficiency. These findings emphasize that functional equivalence must be considered to identify environmentally optimal synthesis strategies [180].

#### 6.5.1. Ecotoxicity and Recycling Challenges in Wastewater and Biosolids

Regarding the food packaging applications, AgNPs showed some concern on their ecotoxicity. Once AgNPs-enhanced packaging is used and disposed of in wastewater streams, AgNPs can enter treatment systems. Pilot studies in real wastewater treatment plants demonstrate that most AgNPs rapidly sulfidize (forming Ag_2_S) and sorb onto biosolids, with less than 1% remaining as free nanoparticles in effluent streams [181]. Despite this transformation, biosolid-enriched soils may experience altered microbial community structure and suppressed ecosystem functions due to residual silver bioavailability, even if the nanoparticles are partly in inert forms. Such fate dynamics underscore the importance of considering long-term accumulation in sludge-amended soils within environmental risk assessments.

AgNPs–polymer materials often take the form of multilayer films or coated composites, complicating mechanical recycling due to mixed-material incompatibility and nanoparticle contamination. Less than 5% of such packaging is successfully recycled in practice, with the rest directed to incineration or landfills. This results in resource loss and potential environmental release of silver at disposal sites. In biodegradable polymers, AgNPs can impair compostability: recent studies reveal that AgCl-embedded nanocomposites reduce soil microbial respiration and retard biodegradation, potentially disrupting soil ecology [182].

At end-of-life, AgNPs embedded in polymer matrices undergo transformation processes depending on pH, redox state, and co-occurring ions. The most common pathways are oxidation to soluble Ag^+^, sulfidation to Ag_2_S (poorly soluble and less bioavailable), and chlorination to AgCl [183]. These species can partition into sewage sludge, surface water sediments, or solid waste residues. Sustainable management strategies include mono-material polymer design that facilitates mechanical recycling, recovery of silver via chemical or electrochemical extraction, and controlled composting/incineration routes with emission capture [184,185]. Together, these approaches can reduce uncontrolled silver release and improve environmental compatibility of AgNP-polymer systems.

#### 6.5.2. Mitigation Strategies and Sustainable Design

The need to reduce the possible harmful effects of AgNPs without compromising their antibacterial functionality has gained researchers’ attention lately. A key avenue involves rethinking how silver nanoparticles are synthesized in the first place. Green synthesis, the biological synthesis of AgNPs, is a promising strategy since, in the reaction, natural and biocompatible compounds are introduced. This approach can eliminate other possible chemical regents, but also dramatically lowers the overall energy demand of the nanoparticle production process [186]. Secondly, the implementation of single-polymer matrices or biodegradable polymers is a strategy which will sustain the fabrication process. The third dimension relates to the release of AgNPs in nature and environmental systems. Targeted post-use strategies, such as pre-validated composting protocols, land application safety studies, and sludge stabilization research, are essential for minimizing unintended nanoparticle release into terrestrial ecosystems. Proactively integrating such considerations during the design phase of the material can help the industry to anticipate and mitigate environmental risks while supporting circular economy principles and regulatory compliance.

#### 6.5.3. Environmental Impact Across the Entire Life Cycle

The environmental impact is not limited to the end-of-life but extends across the entire life cycle, from raw material extraction to disposal. A Life Cycle Assessment (LCA) provides a holistic framework for comparing these impacts to conventional alternatives [187].

Environmental burdens begin with the extraction of silver. Traditional mining is energy-intensive and has significant environmental ramifications, including habitat destruction and toxic emissions. However, a more sustainable alternative is the recovery of silver from electronic waste (e-waste), which aligns with the principles of a circular economy and reduces reliance on virgin mineral extraction [188]. The manufacturing process of AgNPs is also a key contributor to the overall footprint, as it can be dominated by upstream electricity consumption [187,189]. Life cycle studies on nanosilver-enabled products, such as T-shirts, have shown that the choice of production technology can result in a wide range of environmental burdens, particularly in terms of carbon footprint [189].

During the use phase, AgNPs can be released into the environment, particularly from products like textiles during washing [189]. The environmental fate of these released nanoparticles is complex. In wastewater treatment plants, AgNPs are not broken down but rather transform, typically into Ag_2_S nanoparticles, which then settle into sludge [190]. The subsequent environmental impact depends on the fate of this sludge. If used as agricultural fertilizer, the silver can accumulate in the soil, potentially harming soil ecosystems [189]. This highlights the long-term, cumulative nature of environmental risk.

A comparative analysis reveals a complex trade-off between AgNPs and conventional antimicrobials. For example, an LCA comparing nanosilver T-shirts to those treated with triclosan found that the production phase of nanosilver products can have a higher climate footprint. However, the potent antimicrobial efficacy of AgNPs can potentially lead to a reduced need for washing, which could offset the production-phase impact during the use phase [189]. Furthermore, AgNPs may offer an advantage over conventional antibiotics by targeting bacteria in a non-specific way that can reduce the development of antimicrobial resistance, a major global health and environmental concern associated with the widespread use of traditional antibiotics [191].

## 7. Future Perspectives and Opportunities

**Figure 10:**
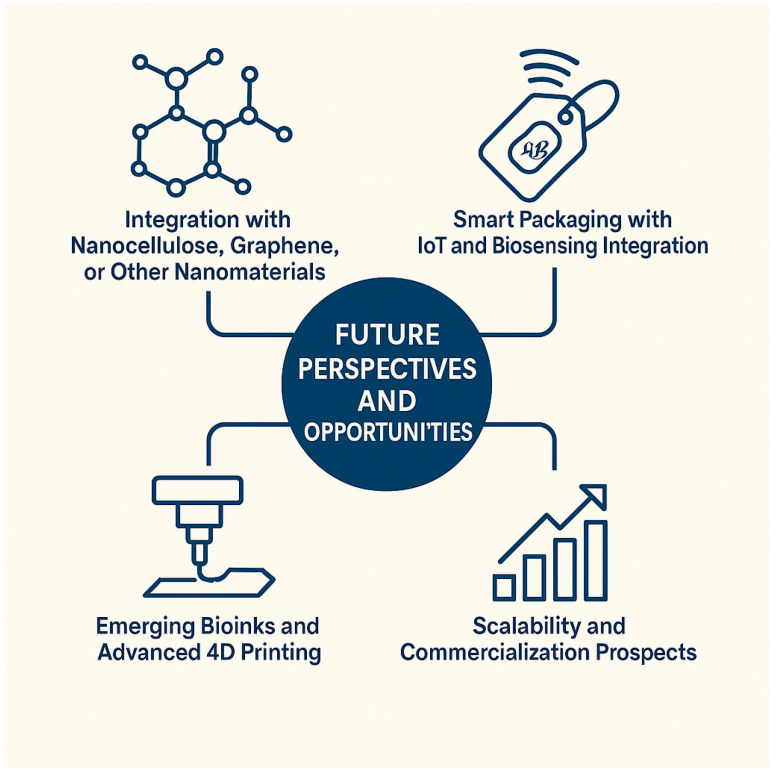
Future directions for AgNPs–polymer systems in biomedical and food applications.

The next generation of AgNPs–polymer systems is being driven by several converging innovations, as they are described in Figure 10.

### 7.1. Integration with Other Nanomaterials

When comparing different antimicrobial nanoparticles, silver nanoparticles are widely recognized for their potent, broad-spectrum efficacy, often showing higher activity at lower concentrations than other metal nanoparticles like copper and zinc oxide. However, all three types of nanoparticles have demonstrated strong antimicrobial effects against various pathogens [192].

The strategic integration of AgNPs with nanocellulose and graphene-based materials represents a powerful multidisciplinary convergence. High-aspect-ratio cellulose nanofibrils or nanocrystals can serve as reinforcing matrices that anchor and stabilize AgNPs while improving mechanical strength and barrier performance. For example, sulphidized cellulose nanofiber coatings embedded with AgNPs applied to paper substrates create dense, uniform films that significantly reduce water vapor permeation, enhance tensile properties, and provide strong bactericidal action against *Escherichia coli* and *Staphylococcus aureus*. In these systems, AgNPs remain well-distributed within the cellulose matrix, releasing nanoparticles gradually and demonstrating minimal cytotoxicity to human colon cell models [193].

Hydrogels composed of nanocellulose and alginate, with in situ-synthesized AgNPs, have shown excellent antimicrobial efficacy, biodegradability, and controlled decomposition in both biomedical and food industries. In food packaging, the study reported by Somsesta et al. showed a decomposition of such system within 15–30 days in soil, making them well-suited to sustainable active packaging, even for poultry products, with shelf-life extension while meeting biocompatibility thresholds (e.g., ≤15 mg total volatile basic nitrogen; 99.99% bacterial inhibition) [194].

### 7.2. Smart Packaging with IoT and Biosensing Integration

Recent trends in packaging technology emphasize not only passive protection but also active monitoring of food quality throughout the supply chain. This dynamic focus aligns particularly well with AgNPs–polymer systems, which can serve as both antimicrobial agents and integral components of smart sensing platforms.

#### 7.2.1. Colorimetric Sensor Arrays

Leveraging the LSPR of AgNPs, colorimetric sensor arrays offer a visually intuitive freshness indication. A recent study utilized agar-embedded AgNPs arrays to detect volatile organic compounds, such as amines and hydrogen sulfide, released during meat spoilage. The system exhibited distinct colorimetric changes in response to 12 key spoilage compounds, offering both qualitative and quantitative freshness assessments without complex instrumentation [195]. Such arrays mimic olfactory detection mechanisms, and are exceptionally low-cost and easy to interpret.

#### 7.2.2. IoT-Enabled RFID/NFC Smart Tags

Radio-Frequency Identification (RFID) and Near-Field Communication (NFC) tags embedded in packaging films present a transformative route for real-time food monitoring. For instance, innovative research demonstrated the use of ammonia-sensitive layers (based on AgNPs-graphene or conductive polymers) integrated into chipless RFID resonators, allowing visual and wireless detection of spoilage gases. These tags can report temperature, humidity, or gas milestones detectable by smartphones or scanners, enhancing traceability while maintaining package integrity [196]. Advancing further, a cutting-edge proof-of-concept design of a battery-free stretchable packaging combining an NFC antenna, gas sensor, and controlled release layer for fish packaging has been developed. The system automatically harvested power from external readers, detected ammonia build-up, and triggered the release of antioxidant and antibacterial agents, effectively extending shelf life by two weeks [197]. This closed-loop, intelligent packaging model exemplifies how AgNPs can be part of hierarchical systems that sense, signal, and respond.

Regarding the challenges, scalability and cost pose significant barriers, with large-scale production of flexible electronics and nanocomposite films depending on affordable conductive inks, such as silver flakes dispersed in polyurethane, and roll-to-roll manufacturing processes [197]. Additionally, the durability of these sensors remains a concern, as they must maintain reliable performance under mechanical stress, humidity fluctuations, and temperature variations during processing and storage. Nevertheless, the integration of AgNPs antimicrobial properties with plasmonic sensing and flexible electronics offers a promising pathway toward the development of packaging systems that are not only protective but also intelligent, responsive, and consumer-oriented.

### 7.3. Emerging Bioinks and Advanced 4D Printing

The evolution of bioinks toward multifunctional and stimuli-responsive behavior has opened new frontiers in advanced fabrication. Incorporating AgNPs within hydrogels or polymer matrices enhances not only antimicrobial performance, but also printing fidelity, mechanical resilience, and responsiveness to environmental cues. A comprehensive review by Chakraborty et al. [198] highlighted how nanoparticles reinforce bioinks in three key dimensions: improving rheological and mechanical integrity for extrusion, introducing bioactivity for cell-material interactions, and enabling responsiveness to external stimuli such as temperature, pH, magnetic fields, or near-infrared light, thereby setting the stage for functional 4D constructs [198].

Smart hydrogel matrices, capable of morphing shape in response to temperature or hydration, can entrap AgNPs to offer programmable release profiles and dynamic structural behavior. For instance, temperature-sensitive poly(N-isopropylacrylamide)-based gels embedded with nanoparticles undergo coil-to-globule transitions, tightly confining particles above the transition temperature and sharply reducing diffusion. This allows on-demand AgNPs release or mechanical actuation [199]. Although direct incorporation of AgNPs into such 4D systems is relatively incipient, the parallels between controlled nanoparticle release and shape morphing make them particularly compatible. By combining responsive polymer backbones with silver nanoparticle functionality, future printable materials could exhibit both shape-transformative behavior and targeted antimicrobial action.

Crafting AgNPs-based bioinks for 4D printing involves balancing multiple factors: maintaining nanoparticle stability and dispersion, tuning rheological properties for reliable extrusion, and programming responsiveness (e.g., thermal, pH). Achieving simultaneous cell compatibility, mechanical strength and print fidelity remains nontrivial, especially for biomedical or biodegradable packaging applications.

Beyond silver, other nanomaterials, such as graphene oxide or carbon quantum dots, enhance bioink performance. Graphene oxide inclusion improves viscosity, electrical conductivity, barrier function, and UV stability, enabling robust extrusion and printing precision while supporting cell viability [200]. Carbon quantum dots have also been used to adjust rheological properties and impart fluorescence for theragnostic applications, although their application alongside AgNPs in 4D printing remains exploratory.

### 7.4. Cost Optimization, Scalability, and Commercialization Prospects

The journey from laboratory-scale development of AgNPs–polymer composites to full-scale commercialization is complex, requiring convergence across material science, industrial processing, economics, and regulatory compliance. While considerable progress has been made in optimizing functionality and safety at the research level, scaling these systems up while maintaining performance, sustainability, and affordability is an ongoing challenge.

Continuous flow systems have emerged as a promising alternative to batch synthesis for producing AgNPs with narrow size distributions and consistent surface characteristics, crucial for integration into polymer matrices [201]. These systems, combined with green synthesis routes utilizing natural reductants like glucose or plant extracts, reduce environmental impact and make nanoparticle production more industrially feasible [202]. Furthermore, incorporation of AgNPs into polymer systems at scale demands compatibility with high-throughput processes such as extrusion, roll-to-roll coating, or spray deposition. Techniques like solvent casting or electrospinning, while useful at the lab scale, are often limited by production speed and solvent handling requirements. As a result, scalable methods such as melt compounding or film blowing, where preformed or in situ-synthesized AgNPs are dispersed into thermoplastics, are being increasingly explored for commercial adoption [203].

The high cost of silver is a critical factor limiting the widespread commercialization of AgNPs-based materials. Therefore, much of the current focus is on reducing AgNPs loading while maintaining or even enhancing antimicrobial efficacy. Studies have shown that silver contents as low as 0.1–0.3 wt% can achieve sufficient antimicrobial protection when synergistically combined with other functional agents like curcumin, essential oils, or plant extracts [204]. The development of composite materials with hierarchical structures, where AgNPs are concentrated near the surface or in functional layers, also improves material efficiency by reducing the total silver required [205].

Economic models suggest that antimicrobial films with silver contents below 20 mg/m^2^ can be cost-effective for high-value food packaging and biomedical applications, especially when positioned as premium products that offer extended shelf life or reduced infection risk [206].

Consumer perception is another key driver of success. While nanotechnology can be viewed with skepticism, studies show that clear labeling, demonstrable benefits, and sustainability claims can increase acceptance [166]. Traceability tools like QR codes or NFC chips embedded in packaging allow consumers to access product provenance, shelf-life data, and recycling information, aligning with the broader trend of smart, connected packaging.

AgNPs are often viewed as costly, but at application-relevant loadings their cost impact can be modest relative to performance gains, provided release is controlled. As of 21 August 2025, the silver spot price is ~USD 1230–1240 per kg (≈USD 38/oz) [207,208]. At typical antimicrobial loadings for polymer matrices (10–100 ppm Ag, supported by recent efficacy/cytotoxicity reports), the silver cost contribution is USD 0.01–0.12 per kg of polymer; even at 1000 ppm it is ≈USD 1.23/kg based on metal value alone [207]. Commercial AgNP formulations introduce processing and margin premiums (illustratively USD 1200–2500 per kg of AgNP formulation, vendor-dependent), but because dose is low, the per-kilogram composite surcharge remains in the cents–low dollars range [209]. By contrast, lower-cost oxide alternatives (e.g., ZnO or TiO_2_ NPs) are often used at 0.3–1.0 wt% to reach comparable antimicrobial durability in polymers; at representative prices (ZnO NPs ≈ USD 115–150/kg; TiO_2_ NPs ≈ USD 190–980/kg depending on grade/quantity), this translates to USD 0.35–9.8/kg polymer, overlapping or exceeding AgNPs’ costs when Ag is dosed ≤100–200 ppm [210,211,212]. Beyond raw materials, economic viability hinges on the following: (i) manufacturing fit (in situ vs. ex situ incorporation, dispersion energy, and drying/annealing steps); (ii) regulatory/testing spend (migration/biocompatibility packages); and (iii) performance durability (longer antimicrobial lifetime reduces total cost of ownership). Thresholds for commercial viability are, therefore, met when Ag loading is kept at ≤100–200 ppm with validated slow-release kinetics, extending service life and offsetting modest material premiums relative to commodity antimicrobial fillers [213].

Scalability must also account for end-of-life management. AgNPs–polymer systems should be designed with recyclability or biodegradability in mind, ideally using mono-material substrates and avoiding toxic crosslinkers or irreversible chemical bonding. Compostable polymers, combined with biosynthesized AgNPs, have shown promise for achieving both performance and environmental compliance [214].

## Figures and Tables

**Table 1:** Overview of AgNPs sizes depending on the synthesis route.

Synthesis Method	Sizes	Key Aspects	References
***Green synthesis***	4.06 nm	Strong antibacterial effect on foodborne pathogens	[52]
***Green synthesis***	40/58 nm	Stronger antibacterial effect was proved for the smallest AgNPs using microscopy	[49]
***Green synthesis***	18 nm	Antibacterial activity confirmed using disk diffusion method: once the AgNPs concentration is increasing, the bactericidal effect is also higher	[53]
***Chemical synthesis***	10/20/75 nm	Antibacterial activity and cytotoxicity showed a higher effect for the smallest AgNPs	[54]
***Chemical synthesis***	5–100 nm	Antibacterial test performed on four different strains showed the bactericidal effect to be size and dose-dependent	[55]
***Physical synthesis***	6.6. nm	AgNPs proved a good antibacterial activity for various bacteria Cancer therapy	[56]
***Physical synthesis***	4/7/40 nm	Incapsulated in a polyurethane membrane, the smallest AgNPs proved the best bactericidal and cytotoxic effect on bacteria and cells	[57]

**Table 2:** A brief comparison regarding AgNPs incorporation in a polymeric matrix.

Method	Advantages	Inconveniences	References
In situ	Uniform NPs distribution; good integration	Requires precise control of reduction	[92]
Ex situ	Tunable NPs characteristics	Poor dispersion; leaching risk	[93,94]

**Table 3:** AgNPs–polymer systems using 3D/4D printing.

Application	Material/Approach	References
Bone regeneration	PLGA/AgNPs scaffolds via direct ink writing	[107]
Wound healing	Collagen/AgNPs patches via UV-assisted bioprinting	[101]
Skin scaffolds	Alginate/PVA/MBGN composite hydrogels	[111]
Biofilm-resistant devices	PDA-functionalized PLA/PCL with AgNPs	[106]
Dynamic (4D) systems	Thermo-responsive hydrogel matrices with AgNPs	[136]

**Table 4:** Regulatory status of AgNPs in food contact materials across key markets.

Region/ Country	Regulatory Agency	Approval Status	Key Conditions/Restrictions	References
European Union (EU)	EFSA/EC	Not approved for general use	Case-by-case approval required. AgNPs not listed in Regulation (EU) No. 10/2011 Annex I. Migration limit: 0.05 mg/kg food; nanospecific data required	[166,167]
United States (US)	FDA/EPA	Not GRAS or FCN-cleared	Requires FDA or GRAS approvement. EPA regulates if marketed as antimicrobial	[169]
Canada	Health Canada	No specific provisions for nanomaterials	Regulated under general food contact standards. Nanosilver is not yet individually assessed or listed	[170]
China	National Health Commission	Not approved	Only bulk silver (Ag^0^) allowed in limited uses. Nanoscale forms are not permitted in food contact polymers	[170]
Brazil	ANVISA	Excluded from positive list	AgNPs not listed in Ordinance 105/2021 for food contact plastic additives	[170]
India	FSSAI	No nano-specific regulation	General material safety standards apply. No official list or migration thresholds for nanomaterials	[170]
Australia and New Zealand	FSANZ	No formal approval	Evaluated case-by-case FSANZ encourages nanospecific data but lacks dedicated regulatory framework	[170]
Japan	MHLW	Cautious stance	Approves substances individually. AgNPs not listed in positive lists under Japanese FCM laws	[170]

## Data Availability

No new data were created or analyzed in this study.
